# Trade-off between jerk and time headway as an indicator of driving style

**DOI:** 10.1371/journal.pone.0185856

**Published:** 2017-10-17

**Authors:** Teemu H. Itkonen, Jami Pekkanen, Otto Lappi, Iisakki Kosonen, Tapio Luttinen, Heikki Summala

**Affiliations:** 1 Spatial Planning and Transportation Engineering, Department of Built Environment, Aalto University, Helsinki, Finland; 2 Cognitive Science, University of Helsinki, Helsinki, Finland; 3 Traffic Research Unit, University of Helsinki, Helsinki, Finland; University of Rome, ITALY

## Abstract

Variation in longitudinal control in driving has been discussed in both traffic psychology and transportation engineering. Traffic psychologists have concerned themselves with “driving style”, a habitual form of behavior marked by it’s stability, and its basis in psychological traits. Those working in traffic microsimulation have searched for quantitative ways to represent different driver-car systems in car following models. There has been unfortunately little overlap or theoretical consistency between these literatures. Here, we investigated relationships between directly observable measures (time headway, acceleration and jerk) in a simulated driving task where the driving context, vehicle and environment were controlled. We found individual differences in the way a trade-off was made between close but jerky vs. far and smooth following behavior. We call these “intensive” and “calm” driving, and suggest this trade-off can serve as an indicator of a possible latent factor underlying driving style. We posit that pursuing such latent factors for driving style may have implications for modelling driver heterogeneity across various domains in traffic simulation.

## 1 Introduction

In any biological system, including human behavior, variability is the norm. Average behavior, “ideal” forms, or population means may capture overall central tendencies, but to understand the complexity and dynamical interactions of most real systems it is necessary to take into account the fact that for any system of interacting agents capable of complex individual behavior—be that birds in flock or motorists travelling down a freeway—real behavior rarely follows a single, simple rule.

Such rules can provide deep insight into system properties, and describe the dynamics at a level of detail that are sufficient for improving understanding and even deriving practical applications. Nevertheless, models that make a simplifying assumption of population homogeneity have their limits. To make progress, the issue of heterogeneity needs to be addressed.

This is the situation currently in the domain of traffic flow modelling—a domain at the intersection of physics (dynamic systems), engineering (transportation systems design) and behavioral science (driver psychology and behavior). Here, we look a the problem of car following (CF), a core behavior in road traffic flow. A deep understanding of CF would have important implications in alleviating congestion and improving traffic system performance. CF is also a fundamental form of vehicle-vehicle interaction that needs to be understood to better design autonomous vehicles that will need to interact with human drivers in mixed fleets.

Traditionally, CF models used in traffic simulation have assumed a fleet of identical drivers, modeled as a simple rule that associates their observations (such as distance to the leading vehicle or current speed) to desired behavior (acceleration or target headway). The validation of model parameters has typically been done by reference to an “average” driver. In recent years, interest in heterogeneous driver modelling has been increasing, with several studies addressing the problem [1–4]. In some commercial traffic simulation software the user is given a choice to include randomness into the parameters of a particular CF model, however the deviations are drawn per-parameter from a parametric distribution [5]. So, although the concept of individual variability in “driving style” has been well established in traffic psychology and engineering for decades [6, 7], these studies rarely overlap with each other or agree on the terms of discussion.

Despite decades of theoretical and empirical work, the field of traffic psychology has failed to converge on a clear and precise definition of the concept of “driving style”, and its psychological underpinnings and relation to important issues such as accident causation remain debated. Work on high-level traffic psychology concepts could benefit from a more concrete approach, such as direct performance measures and experimentally simple scenarios, which are susceptible to rigorous computational modelling.

In this article, we investigate individual variation of car following dynamics, particularly through the relationships between time headway, acceleration and jerk. Previous research has been lacking a theoretical framework to discuss variation in observable CF dynamics, which is something that Sagberg et al. [8] have recently proposed. We take their work as a starting point. Our study differs from past research by drawing from theoretical as well as practical considerations, and providing a controlled simulated driving task, where all participants drive through the same maneuvers for an extended period of time. This kind of data set is good for investigating between-driver differences, as it effectively controls for the differences in the vehicle and the environment, which in this case are identical for all participants.

### 1.1 Driving style in traffic psychology

Within traffic psychology, the question of stable individual differences in how a driver chooses to go about the business of normal everyday driving, “driving style”, has been the subject of extensive discussion for decades. In a comprehensive review of the literature, Sagberg et al. [8] find that the various proposed definitions share three common aspects:

1. driving styles differ across individuals or groups of individuals,
2. driving style is a stable, habitual feature in driving behaviour,
3. driving style is a choice, which many authors equate with a conscious preference.

The authors themselves omit the last criterion of consciousness, and define driving style as “a habitual way of driving, which is characteristic for a driver or a group of drivers”. In this article, we adopt their definition. According to Sagberg et al. [8], driving behaviour includes all the actions taken by the driver in the course of the task. Driving style is a subset of this behaviour, marked by its stability and persistence. We interpret this to mean that—unlike driving skill which is also a stable feature of driving behaviour that varies between individuals—driving style refers exclusively to routine behaviors that the individual repeatedly engages in. For example, panic reactions in a sudden emergency would not be part of a driver’s “style”.

In traffic psychology, driving style is operationalized in terms of a whole host individual preferences, such as speed control, time gap acceptance and thresholds for overtaking or merging into traffic, time headways (e.g. tailgating), and tendency to display emotions to other road users or commit driving violations [8, 9]. The early studies on driving style concentrated more on background factors, such as socio-economic history and personality [6], with more recent studies using both self-report and both qualitative and quantitative observational measures [10, 11].

Sagberg et al. [8] propose a distinction between global and specific driving styles. Here, a global driving style is composed of all the specific styles that comprise the whole of the given driver’s or group’s characteristic driving habits. Specific styles are determined by their domain, e.g. overspeeding, tailgating, or jerky driving.

Empirical research has not yet addressed the question of whether these specific styles are correlated, but Sagberg et al. suggest that they should be: a global style, e.g. “aggressive driving” is a latent factor that will be expressed in specific situations as tailgating, honking the horn, or accelerating hard from traffic lights.

A large part of the research has approached driving style as the search of such latent factors in the hope that they in turn would be correlated with individual crash risk [12]. The goal here trying to find means to identify the “accident prone” type, or other individual differences such as sensation-seeking or aggressive personality traits that predispose people to behaviors that are seen as violations of societally acceptable safe behavior. These underlying traits would then be candidates for psychological intervention. (Driving style is here contrasted with driving skill, which encompasses the perceptual, motor and cognitive skill at which the driver performs the driving task [13]—and could also be a candidate of intervention, namely, driver training).

While questionnaire-based studies to uncover these latent motivations and goals are abundant, there is no consensus yet on the proper taxonomy of global or specific styles. According to Sagberg et al. [8], relevant specific longitudinal control styles are speeding, jerky driving and tailgating. Our primary interest here lies in habits concerning longitudinal control, a specific style, and in particular longitudinal control in car following rather than free flow conditions. Hence, from a traffic psychological perspective, our study focuses on tailgating and jerky driving habits. (The naming itself seems to take a normative stand in the “appropriateness” of these behaviors—we will approach the study habits of longitudinal control purely from a descriptive viewpoint and use the terms merely as legacy terms from traffic safety research). Usually, shorter time headways have been associated with a record of collisions and traffic violations [14, 15].

### 1.2 Driving style classification for advanced driving assistance systems and hybrid vehicles

Several observable measures (usually derived from velocity) have been used for driving style classification for the purpose of developing vehicles themselves, with particular emphasis on cruise control and fuel consumption [16]. “Driving style” in engineering literature is less well defined than in psychology, and the definition varies greatly from paper to paper. Thus what is in this section called driving style does not necessarily conform to the definitions of the previous section. However, it is useful to review some of the findings in this literature, as they relate directly to the measures of interest in this paper.

A report on a Field Operational Test for Intelligent Cruise Control [17] describes a method of driver classification based on time spent in different parts of a normalized range-versus-range-rate space. They use the tail ends of the respective distributions to classify drivers as “ultraconservatives”, “planners”, “hunter/tailgaters”, “extremists” or, if no suitable classification is found, as “flow conformists”. They also rank drivers based on a measure called *confliction*, which indicates time spent in the near region behind a leading vehicle.

Murphey et al. [18] proposed a four-category driving style classifier for the purpose of optimizing fuel consumption. This online classifier looks at a driver jerk profile within a nine-second time window and outputs a style classification to be used by a power management algorithm. The final classification depends on the coeffiecient of variation for a particular time window and a reference value (average jerk of the road type that the vehicle is on). Here, driving style is considered as transient behaviour and as such is not easily related to the concepts of driving style as defined in the previous section.

Bellem et al. [19] have discussed acceleration and jerk as indicators of comfortability in driving with the purpose of developing a driving style for autonomous vehicles. They devised an experiment where maneuver-specific (e.g. *acceleration from non-zero speed*) metrics—such as acceleration, jerk and *quickness*—were shown to differentiate between “comfortable”, “dynamic” and “everyday” driving styles. These driving behaviours were cued by corresponding instruction to the participants. The results suggest that the participant’s conception of comfort is linked to verifiable physical measures.

Most often, the studies mentioned have concentrated on one measure. While these studies are relevant to the discussion of driving style and driver heterogeneity, they lack an attempt to study the interplay of several variables to describe variation in longitudinal control. The categories of classification are important for singular applications, but may be difficult to generalize.

### 1.3 Driver heterogeneity in traffic microsimulation

It’s been acknowledged since the very beginning of car following modeling that different drivers and different vehicles can have substantially different car following dynamics, and that this has effects on the traffic flow [7, 20]. In practice, most effort has been put in finding a single set of parameters for a simulation of homogeneous drivers and vehicles and calibration results are generally reported as a single set of parameters [21]. This is likely due to the homogeneous case admitting to simpler derivation of analytical traffic stability results [1].

However, some recent work has more rigorously studied the heterogeneity and its results on traffic flow. Heterogenous traffic population, especially a mixture of cars and trucks, has been found to explain some of the complex traffic flow dynamics observed in congested traffic [22]. Further studies have found that between-driver differences may be fundamental enough to warrant different model formulations, not only different parameter values, to properly capture the variation [1, 2].

Kim and Mahmassani [3] investigated variability in CF models by looking at the correlations between model parameters when calibrated per driver using the NGSIM dataset [23]. They also show numerically that these correlations should be taken into account when heterogeneous driver populations are generated in a simulation setting. Kim et al. [4] investigated the same issue, and used the same dataset, but instead of studying parameter distributions from independently calibrated datasets, presented a method for directly estimating the distribution by assuming per-driver parameters to follow a multivariate normal distribution. They too found significant correlations between the parameters.

Direct usage of CF model parameterizations has obvious benefits for establishing more realistic parameter distributions for simulation software. However, estimating parameters for modern CF models is a rather complex issue [24], and results based on them are dependent on both the model and the estimation method used. The model specificity of the parameters makes it rather difficult to generalize the results to e.g. traffic psychological theories and studies, which usually discuss the driving process using more direct physical measures.

Also, many of the recent results are based on relatively short trajectories recorded in normal traffic, which makes it difficult to estimate how much of the parameter variance and correlation is due to driver heterogeneity, and how much due to vehicle and driving situation heterogeneity. Pariota et al. [25] measured a large and representative sample of drivers using an instrumented car, thus controlling for the vehicle-specific variation. They found substantial variation between and within different drivers’ car following behavior, operationalized as estimated equilibrium time headway and spacing. The between driver variation was found to be substantially larger than within driver. However, due to lack of controlled environment, their dataset didn’t admit to a more detailed study of per-driver driving style measures and their correlations.

### 1.4 Aims of the study

It has been well established that individual differences in longitudinal driver behaviour occur, and that they have an effect on traffic flows and are correlated to risky road behavior. While the measures of acceleration, jerk, and time headway have been used to parameterize driving style in particular contexts and for specific reasons, most studies use only one of those for classification and have no theoretical framework to build on or generalize the results in a comparable way. Following Sagberg et al. [8], a hypothesis of their correlatedness can be formulated, assuming one or more latent factors which underlie different driving styles. Furthermore, correlations between CF model parameters have been observed, which can be taken to suggest that this might be the case for physical measures as well.

We tested this hypothesis in a driving simulator, where 15 drivers drove in controlled car-following conditions for an extended period of time. Measuring acceleration, jerk and time headway, our aim was to discover the connection between these measures to provide an tentative account of the physical characteristics of longitudinal driving style.

## 2 Methods

### 2.1 Participants

15 participants (5 Male, 10 Female, mean age 31 years, SD = 8.26) took part in the study. The participants were required to hold a valid driver’s license for a passenger vehicle (Finnish driving license class B), and have over 30 000km of self-assessed driving experience or to have held a driver’s license for more than 5 years. Participants were recruited through personal contacts and university mailing lists.

An informed consent to participate was obtained in writing from each participant before starting the experiment. This was done in accordance with the instructions of the ethics committee in the form of a fixed-format consent form explaining the purpose of the study, the procedure, and intended use of the data (for scientific purposes only). The study was conducted following the research ethical guidelines of Finnish National Advisory Board on Research Ethics and Helsinki Ethical Review Board in the Humanities and Social and Behavioural Sciences. As per the guidelines, ethical review for the experiment was waived, as the experiment didn’t include any of the criteria that warrant for such review. Apart from e-mail addresses, no identifying information was gathered in the study. The e-mail addresses were removed from the data set in the first step of preprocessing and were stored in a separate file only on the data logging computer and one workstation.

After giving consent, the participants filled an electronic questionnaire with nine questions. This included information about their age, gender, driving license class, driving experience and habits of playing video games. The majority, 73% only had a passenger car driving license (Finnish class B). The subject’s self-reported lifetime driving experience varied between 1000–300 000 km, with gaming experience ranging from “none” to “daily”. The detailed background information can be found in the S1 Table in the supplement.

### 2.2 Driving simulator

The driving simulator was located in the Traffic Research Unit, Faculty of Behavioral Sciences, Uni. Helsinki. The experimental space was a small room which was closed at the time of experimentation. The simulator consisted of a 55” screen (LG 55UF851V), driving game controller with a steering wheel and pedals (Logitech G25) and an adjustable driving seat (Playseat Evolution Alcantara). The seat could be manually adjusted with sliders, allowing the participant to choose a comfortable distance from the wheel. The exact viewing distance to the screen depended on the participant’s body size and preference, but was generally within 80–90 cm. This created an approximately 70 degree viewing angle to the 55” screen, which is the same as the virtual angle of view used in the software. Simulated engine noise was presented through the screen’s speakers. For a more detailed description of the driving simulator, see [26].

The driving simulator software was developed in-house and is available under an open source license [27]. The parameters of the simulated vehicle dynamics were decided by informal pilot testing to give a comfortable compromise of good controllability but not overly nervous responses.

### 2.3 Experimental design

The experiment consisted of a training phase and a car-following phase. The purpose of the training phase was to familiarize the participant with the dynamics and dimensions of the virtual vehicle. In the car-following phase all visual dials were removed from the screen and the participant had to judge his own speed with no objective speedometer. A braking light was present in the leading vehicle. Steering was disabled for the entire duration of the experiment, and the participant controlled the vehicle only through the gas and brake pedals.

**Fig 1:**
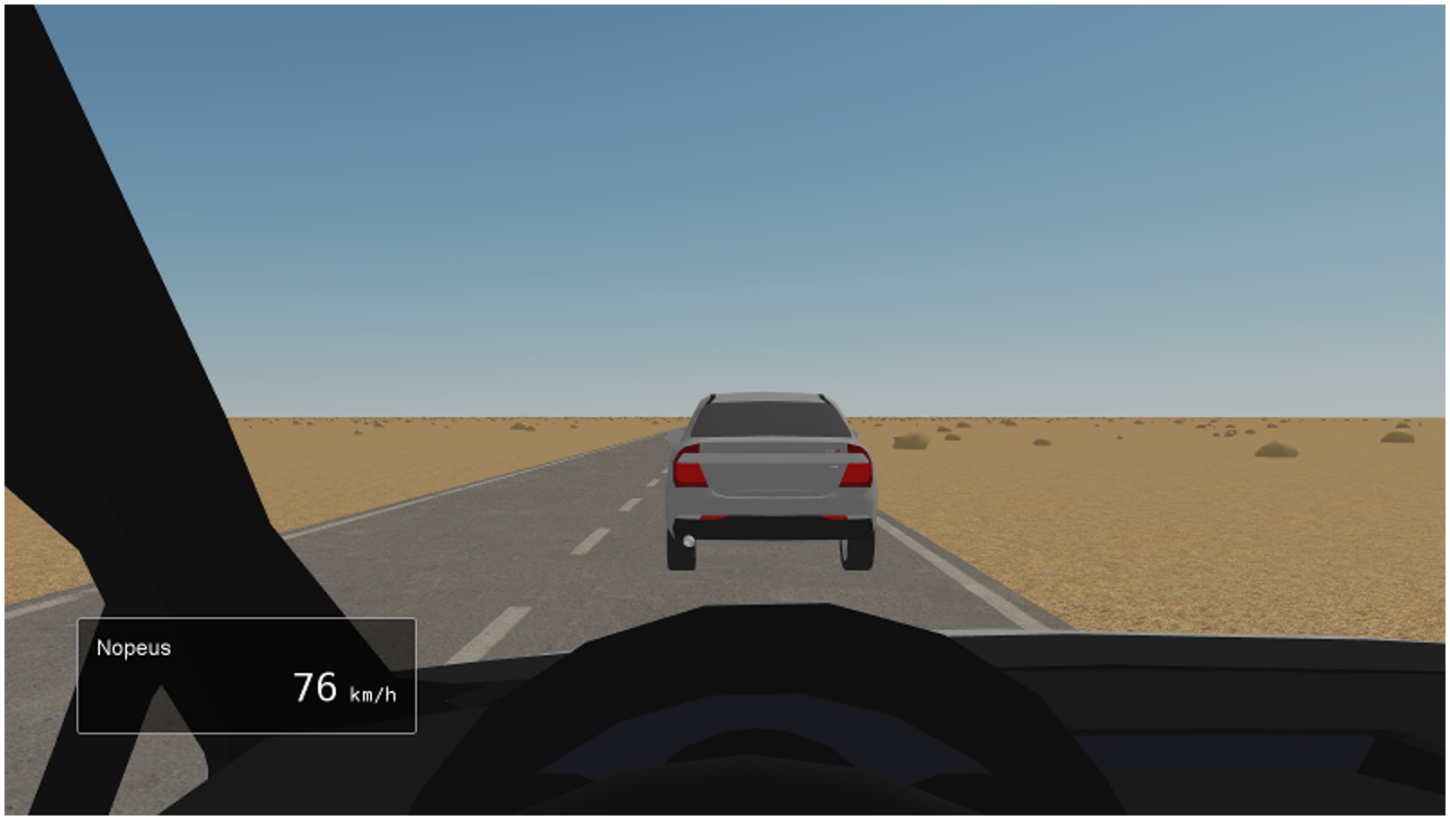
Sample screen from the training phase.

The driving environment consisted of a single road with two lanes, separated by a center line. The right-hand lane was used for the experiment, with no oncoming traffic present. The landscape consisted of a sand-like ground texture, with some rocks scattered in the scenery. The textures and rocks provided for an optic flow pattern when the subject vehicle was moving, which aided the participants in determining their own speed. See screenshot in Fig 1.

In order to create a car-following scenario, a speed profile for the leading vehicle was decided. Four speeds were chosen as the targets of the leading vehicle:

- *10 km/h*: Very slow. Crawling speed.
- *30 km/h*: Slow. Residential area speed limit.
- *50 km/h*: Moderate. City limit.
- *80 km/h*: Fast. Basic speed limit for non-motorway traffic in rural areas.

The entire course of the car-following phase consisted of a series of *blocks*. Each block started and ended in a standstill, with a unique permutation of the four leader target speeds in between. The entire car-following phase was formed by 24 blocks, representing the 24 possible permutations of the four target speeds. Order of the blocks was randomized for each participant. To prevent participant fatigue, the series was divided into four block segments, in between which the participants were allowed take a short pause.

The throttle and brake of the leading vehicle were controlled by a proportional-integral controller, which was given an per-transition independently randomized target acceleration/deceleration from the set of 1.5*m*/*s*^2^, 4.0*m*/*s*^2^ and 7.0*m*/*s*^2^.

As the leading vehicle reached a particular non-zero target speed, it stayed at that speed for 20 seconds with a ±5 second random deviation. When the vehicle came to a full stop between the blocks, it remained stationary for 5 ± 2 seconds after the participant vehicle had come to a full stop. The stochasticity was introduced to prevent the participant from being able to guess when the next accelerating or decelerating maneuver was required.

The duration of the entire experiment was within 46–56 minutes and the car following task analyzed amounted to approximately 30 kilometers of driving per participant.

The drivers were instructed to drive normally, “like driving on a highway”, and that while colliding was not forbidden, if a crash should occur they would have to drive the current segment again and the experiment would be prolonged accordingly. Only one collision in total occurred in the experiment, and the segment where it occurred was omitted from the analyses.

Although the participants could not see other cars than the one ahead, they were told that they were driving in the middle of a queue, and that this was the cause of the fluctuation in traffic. Finally, the participants were instructed to keep the sort of gap which “feels appropriate” and that there was no chance of overtaking.

### 2.4 Analysis

The car following dynamics were operationalized using acceleration, jerk and time headway for each subject. For acceleration and jerk we use their *absolute* values averaged over time. For time headway, we use a *geometric mean* in order to curtail the influence of the “long tail” of the distribution. For brevity, these variables are simply referred as *mean acceleration*, *mean jerk*, and *mean time headway*. The subject means are calculated from the entire length of the experiment.

Acceleration is also analyzed as a function of time. For this analysis, we treat the different speed changes (e.g. from 50km/h to 80km/h) separately, and refer to them as *transitions*, or by their endpoints in brackets, e.g. {*50, 80*}. The beginning of a transition is marked by the onset of a new target speed for the lead vehicle. We consider only the first 15 seconds from that moment. We also further categorize the individual pass-throughs of these transitions based on the time headway the driver held at the start of the transition, dividing them into four quartiles. The first quartile contains the shortest observed time headways and the fourth one the highest.

**Fig 2:**
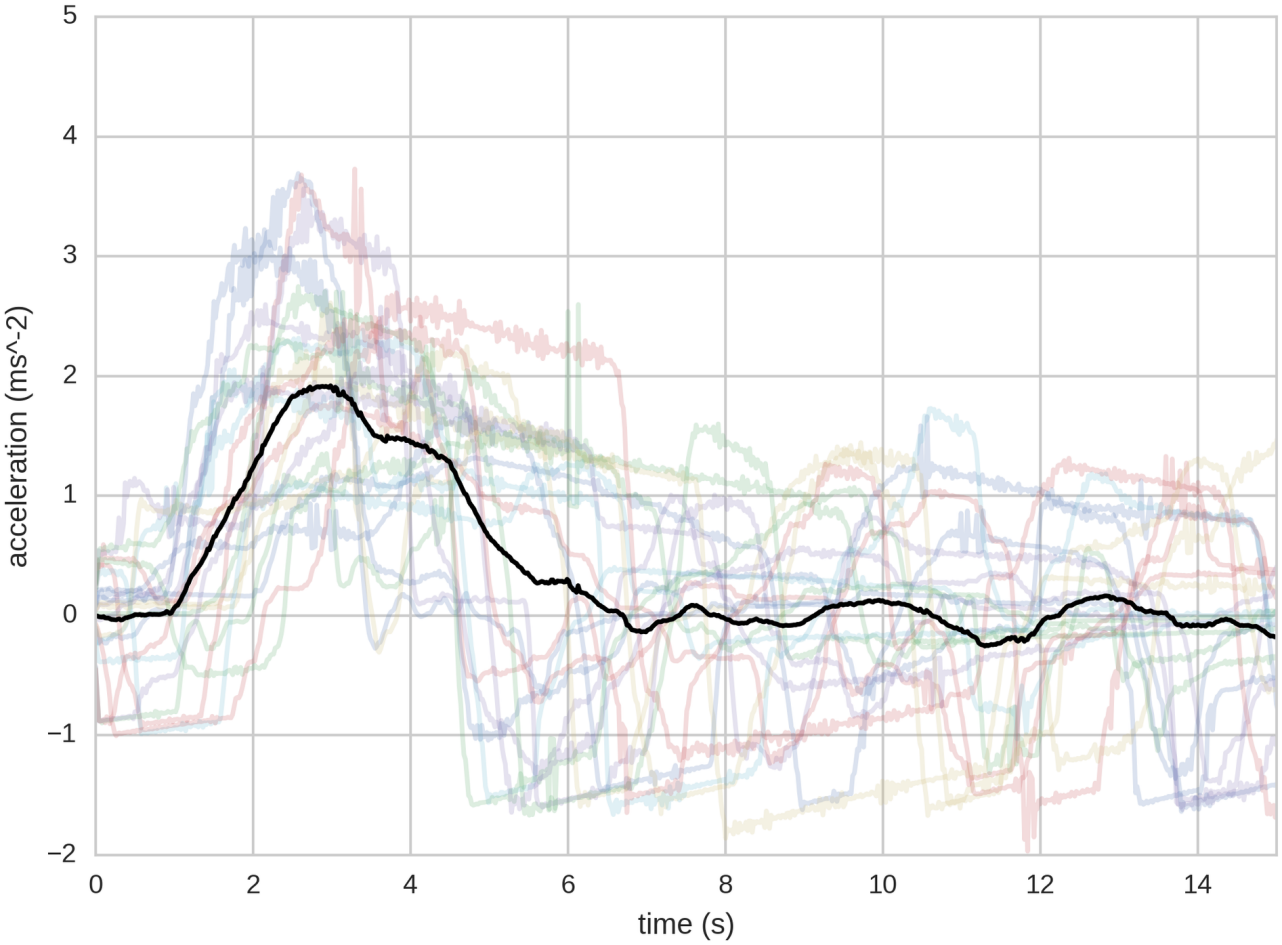
The first quartile of transition {30,50}, i.e. with a start time headway < 2.16*s*. In colour: the raw acceleration data from 23 pass-throughs belonging to this transition and quartile. Black: the average acceleration profile of the pass-throughs. Zero point is the moment when the lead car changes it’s target speed.

The quartile-clustered time series are re-sampled and averaged to provide a graph that represents the average response to lead vehicle speed change. This process is illustrated in Fig 2, representing the first quartile of transition {30,50}, with raw data shown in the background.

After inspecting the linearity between the subject means for mean acceleration, mean jerk and mean time headway, we use simple linear regression to model the connections. To avoid multicollinearity in having several correlated predictors and more importantly, to show that the measures can be adequately represented with a single factor, we use Principal Component Analysis (PCA) to validate the central claim of the paper.

## 3 Results

**Fig 3:**
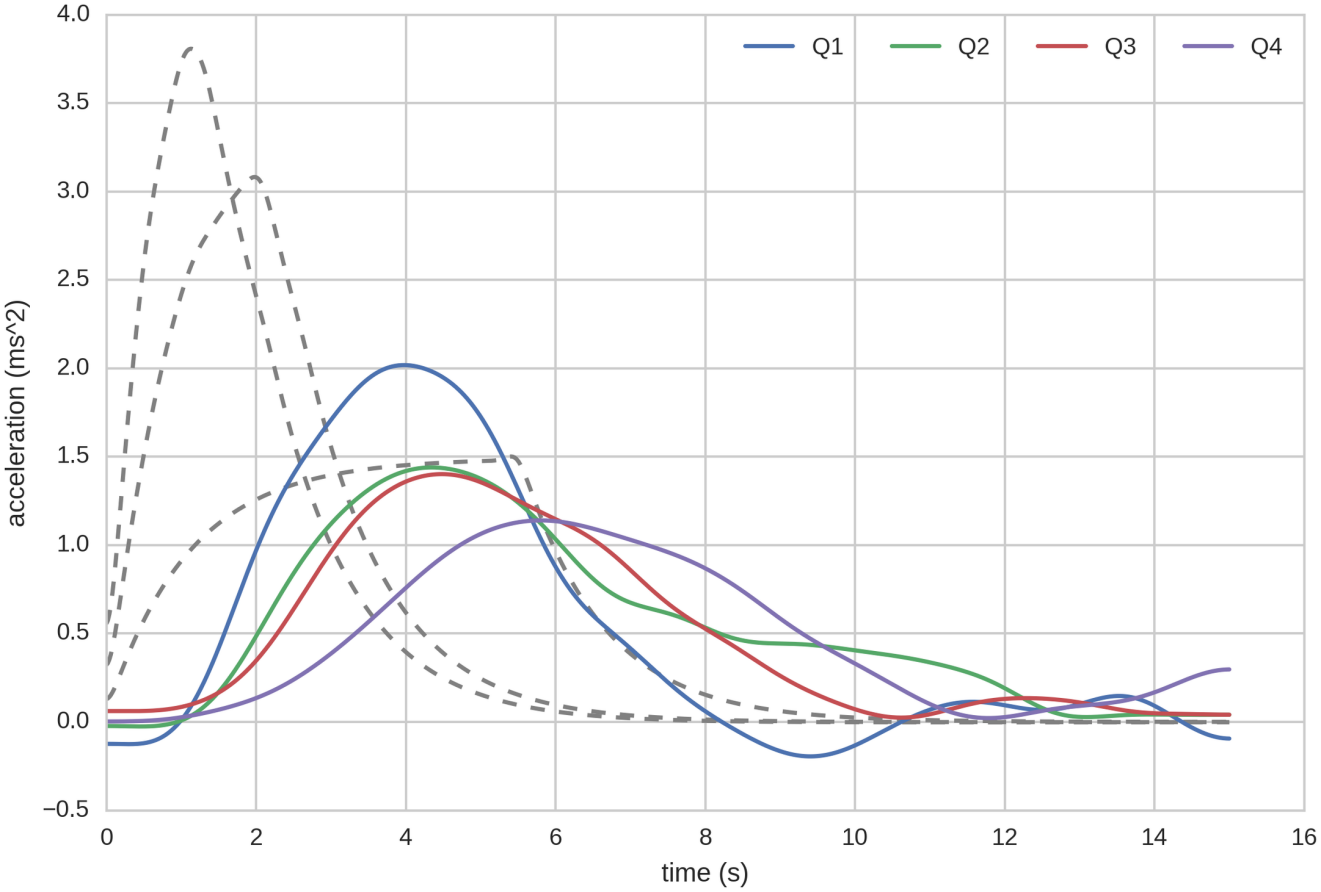
Average acceleration of the following vehicle in speed transition 50km/h to 80km/h. The coloured curves represent quartiles based on time headway at the start of the transitio. The dotted lines are the three different acceleration profiles of the lead vehicle. For this transition, the quartiles (in seconds) correspond to *Q*1 < 1.98, *Q*2 = {1.98, 2.90}, *Q*3 = {2.90, 4.12}, *Q*4 > 4.12. All curves have been smoothed by a Gaussian filter, with sigma corresponding to 0.5s. For other transitions see supplementary figures.

**Fig 4:**
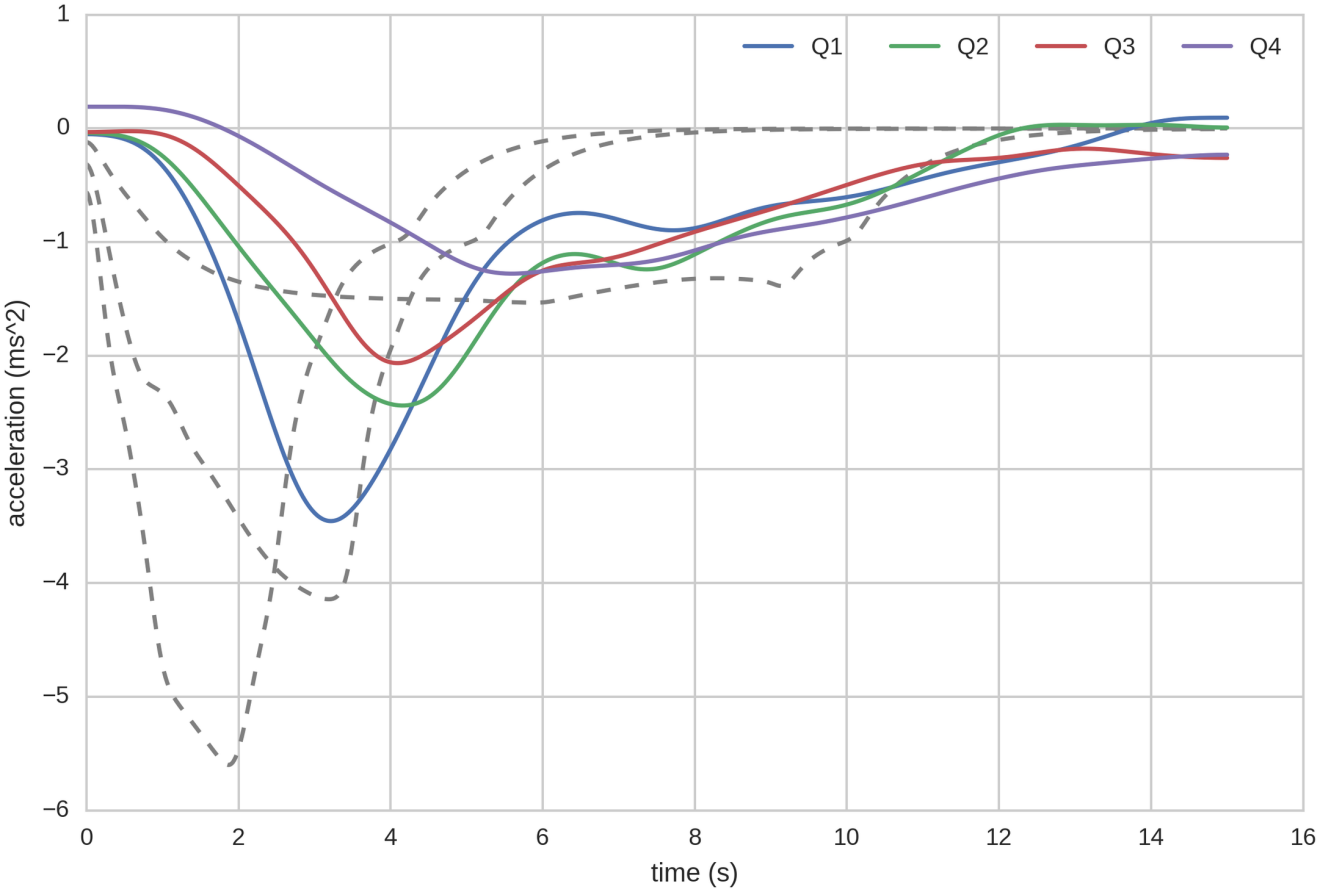
Same as Fig 3, in a decelerating transition (from 80km/h to 30km/h). The quartiles (in seconds) correspond to *Q*1 < 2.12, *Q*2 = {2.12, 3.41}, *Q*3 = {3.41, 5.31}, *Q*4 > 5.31. For other transitions see supplementary figures.

Average acceleration of the following vehicle shows systematic variation when categorized into quartiles by time headway. From the averaged time series in Figs 3 and 4 it can be seen that generally the acceleration response decreases in magnitude as the time headway grows. This also prolongs the response, leading the vehicle to catch up more slowly with the new stable velocity, thus reducing jerk. Only two examples are shown here; the remaining speed transitions can be viewed in the Figs A-L in S1 File, in the supplementary information.

**Fig 5:**
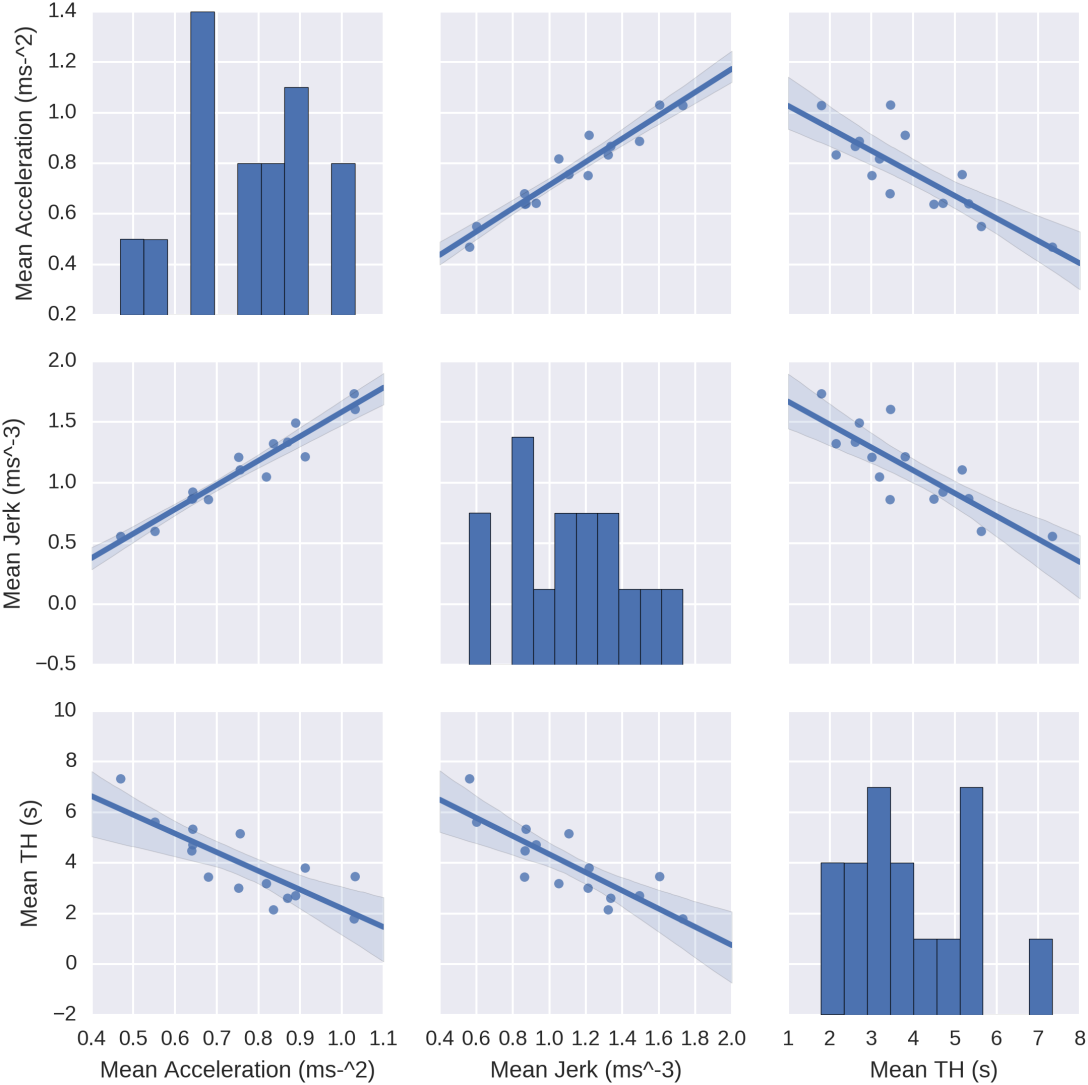
A pair plot of the relationships between mean acceleration, mean jerk and mean time headway and their distributions. The dots represent subject averages, with frequency distributions of the measures on the diagonal.

**Fig 6:**
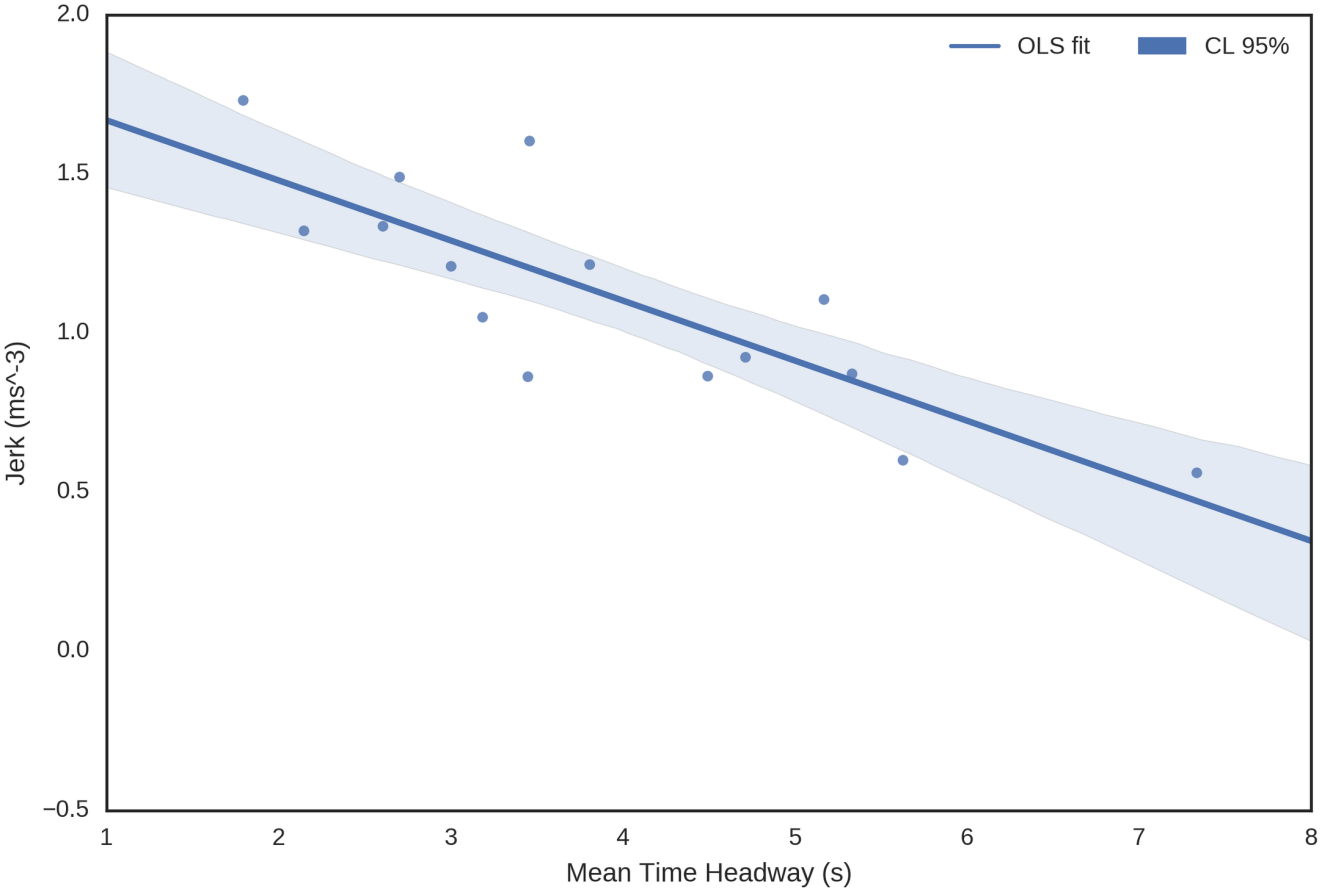
Enlargement of the third panel in the middle row of Fig 5, showing mean time headway vs. mean jerk. The dots represent subject averages. Additional figures, in which data is divided to accelerating and decelerating conditions is provided in the supplements (S1 Fig).

On subject mean level, the results show a clear relationship between time headway, acceleration and jerk. From Fig 5 it can be seen that the measures correlate with each other and the correspondence is highly linear. An enlarged view of mean time headway vs. mean jerk is provided in Fig 6.

Quantitative regression results for acceleration and jerk vs. time headway are shown in Table 1. From Fig 5 and the tabulated results it is evident that each measure predicts the others well, as mean time headway explains 66% and 68% of the variances of mean acceleration and mean jerk, respectively. The highest correspondence is between mean acceleration and mean jerk, with *R*^2^ = 0.92. Slopes of the regressions are significant at *p* < 0.001.

**Table 1:** Regression results between direct measures.

	Intercept *a*	Slope *b*	*F*	*R*^2^
Mean time headway vs. mean acceleration	9.59	-7.39**	25.01**	0.66
Mean time headway vs. mean jerk	7.93	-3.59**	27.46**	0.68
Mean acceleration vs. mean jerk	0.25	0.45**	151.9**	0.92

**p < 0.001

In order to test whether the measures can be reduced to a single dimension, a Principal Component Analysis (PCA) was performed. The PCA reveals that the first component explains 91% of all between-subjects variation in these measures. The second component explains 7%. This suggests that most across-driver variation in time headway, acceleration and jerk can be attributed to variation in single latent “driving style” factor. Details, including loadings, of the PCA can be found in the supplements (Tables A-C in S2 File).

Lastly, we investigated correlations between the physical measures and background information. The correlations are generally small, with the largest ones found between age and mean jerk, mean time headway and the first PCA component (PCA1). Correlation between the second PCA component (PCA2) and driving distance in the past year also reached statistical significance at CL 95%. Full results can be seen in Table 2. Gender differences for PCA1 are not significant in our sample (Mann-Whitney *U* = 20.0, *n*_1_ = 5, *n*_2_ = 10, *p* = 0.29).

**Table 2:** Spearman correlations between measures and background measures. Driving distances are self-reported.

	Lifetime driving distance	Driving distance in the past year	Age	Gaming frequency
Mean time headway	-0.20	-0.28	0.53*	-0.12
Mean acceleration	-0.01	0.11	-0.48	-0.10
Mean jerk	0.04	-0.06	-0.52*	-0.07
PCA Component 1	0.12	0.01	0.53*	<0.01
PCA Component 2	-0.33	-0.52*	-0.04	0.24

*p < 0.05

## 4 Discussion

The results show a clear trade-off between jerky driving and time headway for the participants. On average, drivers opt either to match the leader’s speed at close distance, necessitating more intensive maneuvers when the leading vehicle accelerates or decelerates, or to stay further behind and regulate their speed in a more “calm” manner. This main finding supports the notion that the indicators of jerky driving and tailgating as outlined by Sagberg et al. [8] are reflecting a common factor. This is in line with their assumption of correlatedness of specific driving styles.

We propose that the correspondence between time headway and jerky driving reflects a trade-off that can be interpreted as an “intensity-calmness” parameter of driving style, i.e. the psychological component. We prefer to use the term “intense” instead of “aggressive”, as aggressiveness in traffic psychology literature most typically refers to an emotional state or trait, characterized by feelings of anger and socially hostile behavior. Instead, intensity is used to describe only the observed driving behavior, and may be a product of several psychological mechanisms.

The latent structure of any global driving style is at present unknown, as shown by the variable results of questionnaire-based studies [11, 12]. Questionnaire-based studies reveal only things that the participants can introspect and wish to state, and if the results seem contradictory, then it is best to turn to more objective measures. In any case, they are more easily put to use by traffic simulation. It is however also important to engage in discussion about the underlying psychological mechanisms concerning driving style.

The influence of driving experience to our measures was found to be small or non-existent, which could indicate that the differences are not a result of skill difference, but rather a learned preference. Moderate correlation between the second principal component and self-reported driving experience could be hypothesized to indicate differences in skill leading to “deviation from the trade-off line”, but our participant sample isn’t large enough to make such conclusion.

A similar caveat should be recognized for gender and age, as our sample was predominantly female (66%) and fairly young (mean age 31 years). Both variables are known to affect driving behavior, with unsafe attitudes and risky driving often associated with males and young people [15, 28]. While we found moderate correlations between age and the physical measures, including the first PCA component for “intensity”, the evidence is not strong enough to indicate a robust connection. Replicating the study with a larger, more diverse sample might reveal a more convincing result. The data does not support evidence of gender differences; scatter plots where participants have been differentiated by gender can be viewed in the S2 and S3 Figs in the supplement.

### 4.1 Psychological mechanisms of driving style

One possible contender for the underlying mechanism for the trade-off is a difference in “mental effort” that drivers allocate to the driving task. A car following study with very similar setting to this article found that drivers strongly adapt their headway to attentional demands [26], with similar links between headway control and attentional impairment having been suggested earlier [29, 30]. To consistently keep a short headway, the driver has to track the leading vehicle’s dynamics quite accurately, as otherwise the headway drifts to either a larger value, or more importantly, leads to a crash. Conversely, with a longer headway, or “time margin”, it takes larger deviations from the leading vehicle’s speed for the margin to get too small. Closer tracking of the leading vehicle quite clearly demands more effort. In car following model literature such trade-off has been established from the very beginning in that higher “sensitivity” (acceleration magnitude) requires more “attention” (shorter reaction time) if the driving is to stay stable [7].

The theoretical discussion of speed control in traffic psychology has largely centered around *risk* and the mechanisms that allow drivers to keep the risk level low enough not to crash, while accepting some risk in order to accomplish speedy travel [31]. In a later synthesis by Fuller [32] the concept of risk was replaced by analyzing the driving task as a trade-off between “driving task demands” and “driving capability”. In our experiment the task’s demand can be largely reduced to leading vehicle behaviour and headway control, and the results can be interpreted that drivers indeed modulate the task demands according to their driving capability. Furthermore, in this interpretation the capability doesn’t seem to be dominated by driving skill, but a choice to allocate less mental and/or physical effort to the task.

The theories of risk which aim to explain how such trade-off gets established have somewhat different views on the role of risk in speed control. In what are usually called “target-risk theories” drivers continuously assess the risk in the situation and adapt their behavior to stay at a some subjective non-zero risk level [33–35]. In our experiment this would mean that the drivers select a risk level, which is kept constant by continuously adapting the headway.

The “zero-risk theory” disagrees with this view and argues that drivers habituate to different preferred safety margins (such as time headway or time to line crossing), which become “normal” for them, and any crash risk associated with the particular habitual choice of margins will not be subjectively experienced [36, 37]. An extension of the zero-risk theory [38] further notes that while keeping safety margins within a habituated “comfort zone”, drivers also control accelerations within comfortable limits. In this framework, the space and time margins and accelerations the participants opt to maintain in our simulator experiment could reflect these habitual values.

The heterogeneity among driver-vehicle systems in terms of longitudinal speed control could and should be decomposed into a “psychological component” (probed by our experiment where the vehicle dynamics were identical for all participants), a “vehicle” component (e.g. differences between truck and passenger car driving) and an “ambient” component (visibility, slippery roads). This psychological component would correspond to what in traffic psychology is referred to as driving style.

### 4.2 Implications for traffic simulation

The suggested implication that this discussion has on modelling heterogeneous drivers for traffic simulation is simple: With a more general description of inter-driver differences, one can begin to formulate model parameters which affect driver heterogeneity across different maneuvers.

It is by now well known that driver heterogeneity affects traffic flow [1, 2, 22]. Some recent work has also shown that car following model parameters correlate when calibrated per-driver and that this covariation should be taken into account when modeling driver heterogeneity in simulation studies [3, 4]. This is an important and interesting finding and will hopefully be reflected in future model development.

The usual approach to estimating the parameter distributions has been to calibrate car following models per-driver to trajectory data, which has practical benefits, but the results are problematic to generalize across models and data sets. Establishing a more parsimonious and “model independent” driving style parameterization could be used to generalize driver heterogeneity results across models and across traffic situations. We propose that, alongside model-specific parameter studies, driver heterogeneity should be also studied using direct measures in order to establish more general, quantitative understanding of how driver, vehicle and situation dependent factors are exhibited in more specific driving tasks.

Although providing strong and perhaps more generalizable results, the clear downside of direct measures is that they do not translate to modelling efforts in a trivial manner. To be usable in microsimulation models, future work should study how the driving style can be systematically related to such mechanistic models and their parameterizations. Also the global driving style hypothesis predicts more generally that the style should be reflected in variety of driving behavior, such as gap acceptance, curve negotiation and lane changing tendencies.

### 4.3 Limitations of the study

In general, the veridicality of a driving simulator can not be guaranteed without comparison to on-road studies. Nevertheless, our experiment was kept at minimal complexity and the participant was not presented with extra tasks or more information that is available to a driver of a real vehicle. In addition, the task was framed by the instruction to resemble real driving to prime the participant into using prior knowledge about driving on a highway.

Nevertheless, the possible influence of simulated rather than real driving to the results can not be ruled out. In understanding the task, drivers may adopt a different mindset than in the real world. Video games often require the player to find an optimal solution to the task at hand, and the simulator could have cued behavioral patterns more reminiscent of gaming experience. If, contrary to instruction, the participant understood the task to be “follow the leader as closely as you can”, the result might end up in the the small-gap, high-jerk part of the scale. Conversely, if the participant is trying to optimize for not having to stop or slow down and reasons that the leading vehicle behaves erratically, an unrealistically large-gap, low-jerk strategy might occur. However, the dispersion of the participants along the trade-off axis is quite even, with little extreme behaviour observed. The described behavioral extremes are also valid for real driving.

The trade-off we observed in our experimental setting concerned between-subject variation, and can’t provide information on whether such trade-off occurs within-subject, for example in response to hurry or difficult driving conditions.

We also acknowledge that the magnitude of accelerations and decelerations displayed by the simulated car may be excessive compared to real driving. Because the participants sat still in a closed room, they did not experience a vestibular or somatosensory response, which are produced by real-world accelerations of the body. The kinaesthetic sensations are highly important in judging one’s own acceleration in real driving, to the extent where decoupling visual and vestibular stimuli may cause motion sickness.

While we believe that the sample size is sufficient in demonstrating the existence of a relationship between choice of acceleration, jerk and time headway, it is possible that a larger sample may have revealed more robust correlations between the observed measures and background information, e.g. driving experience. It is unlikely that the size of the sample is adequate for making population-level estimates of the parameters of the trade-off line.

## 5 Conclusions and future directions

In this study we have demonstrated a clear relationship between time headway, acceleration and jerk at the subject level. If this dependency truly serves as an indicator of “global driving style”, as has been discussed, it would be useful to investigate whether it extends to other domains. Visual sampling rate might be a good candidate for future investigation, as closer following distances make it increasingly risky to drive without paying attention to the leader. Considering visual sampling behavior also raises the question of lateral control. Most models of visual control of driving deal mainly with lateral control (for reviews see [39, 40]). This experiment did not have lateral control, but including it in the future could help in integrating visual control models and car-following models. And should the global driving style of an individual turn out to extend to lateral control, lane changing, or curve driving, then this would be an interesting and important result, bringing further integration between the engineering and traffic psychology literatures.

We did not try to correlate the “intensity” of driving style to more traditional psychological measures such as personality, sensation seeking, extraversion or self-reported driving incidents or accidents. This would be an interesting aspect to study in the future, as these connections have been long pursued in traffic psychology. As noted above, it seems plausible that a connection between driving intensity and accident risk exists, especially if coupled with attentional variation or mental effort. It is probable, however, that a detailed explanation of accident risk would need to take into account the driver’s skill as well as driving style—habitual driving patterns alone are likely not sufficient for accurate prediction of risky driving. Even so, we would contend that at the very least, directly observable behavioral measures can provide new information and ultimately contribute to predicting risky behavior.

Another interesting question to study would be the effect of both situational factors and the vehicle with respect to these results. One might presume that participants might change their behaviour if pushed to “hurry” or “slow down”, or do anything else which constricts their freedom to self-determine the task.

Perhaps the largest undertaking is, however, to establish a clear connection between direct measures and the parameters of CF models. Given the potential benefits of looking at direct measures, this could include reformulating the models, or at least demonstrating that the simulated driver population reflects known relationships and obeys their boundary conditions.

In summary, this article has made an effort to tie together discussions of driver variability in both psychology and engineering, and demonstrated that relevant measures in longitudinal speed control vary together—supporting the idea that one or many latent variables underlie habitual driving.

## Supporting information

S1 Table Participants’ background information. Table describing the relevant background information collected on the 15 participants. Driving experiences are self-reported estimates, with 8 discretized categories for lifetime experience (from “Less than 1000 km” to “Over 1 000 000 km”) and 9 categories for the last 12 months (from “None” to “Over 50 000 km”).
(PDF)

S1 File Figs A-L. Per-transition average acceleration figures. For completeness, we provide figures for all the per-transition average accelerations.
(PDF)

S2 File Tables A-C. PCA details. Details of the Principal Component Analysis for variables containing subject averages (N = 15)—mean acceleration, mean jerk and mean time headway. They include eigenvalues, eigenvectors and the loadings for each component.
(PDF)

S1 Fig Jerk vs. time headway—Accelerating and decelerating case. These figures represent the linear fit for the accelerating and decelerating cases for the subject averages of jerk and time headway. The lines are Ordinary Least Squares (OLS) fits along with their confidence intervals at CL 95%.
(PDF)

S2 Fig Mean jerk and mean time headway with gender. Scatter plot showing mean time headway on the x-axis and mean jerk on the y-axis. Male participants in orange, females in green.
(PDF)

S3 Fig First two PCA components with gender. Scatter plot showing the first two PCA components, with gender of the participants differentiated by colour. Should the two groups form distinct clusters, one would be able to see them here. No such pattern arises with this amount of participants.
(PDF)
