# Supplementary material for: Trade-off between jerk and time headway as an indicator of driving style
Source: PLoS One. 2017 Oct 17;12(10):e0185856. doi: 10.1371/journal.pone.0185856 (PMC5645088; doi:10.1371/journal.pone.0185856)
Supplement: S1 Table — Table describing the relevant background information collected on the 15 participants. Driving experiences are self-reported estimates, with 8 discretized categories for lifetime experience (from “Less than 1000 km” to “Over 1 000 000 km”) and 9 categories for the last 12 months (from “None” to “Over 50 000 km”). (PDF) [file pone.0185856.s001.pdf]

S1 Table. Participants' background information

ID	Age	Gender	Driving Experience (lifetime)	Driving Experience (last 12 month)	Gaming Frequency
01	27	female	300 001 – 500 000	1000 - 5000	monthly
02	29	male	100 001 – 300 000	5001 – 10 000	weekly
03	31	male	300 001 – 500 000	15 001 – 20 000	weekly
04	30	female	10 001 – 30 000	< 1000	daily
05	23	female	30 001 – 100 000	5001 – 10 000	ex-player
06	34	female	10 001 – 30 000	< 1000	weekly
07	24	female	100 001 – 300 000	1000 - 5000	none
08	26	female	10 001 – 30 000	< 1000	none
09	25	female	30 001 – 100 000	5001 – 10 000	monthly
10	58	male	300 001 – 500 000	10 001 – 15 000	none
11	35	female	300 001 – 500 000	20 001 – 30 000	ex-player
12	34	female	30 001 – 100 000	5001 – 10 000	none
13	31	female	300 001 – 500 000	20 001 – 30 000	monthly
14	29	male	100 001 – 300 000	5001 – 10 000	daily
15	23	male	30 001 – 100 000	15 001 – 20 000	daily
Mean	30.6				
SD	8.26				
