# Supplementary material for: Trade-off between jerk and time headway as an indicator of driving style
Source: PLoS One. 2017 Oct 17;12(10):e0185856. doi: 10.1371/journal.pone.0185856 (PMC5645088; doi:10.1371/journal.pone.0185856)
Supplement: S1 File — For completeness, we provide figures for all the per-transition average accelerations. (PDF) [file pone.0185856.s002.pdf]

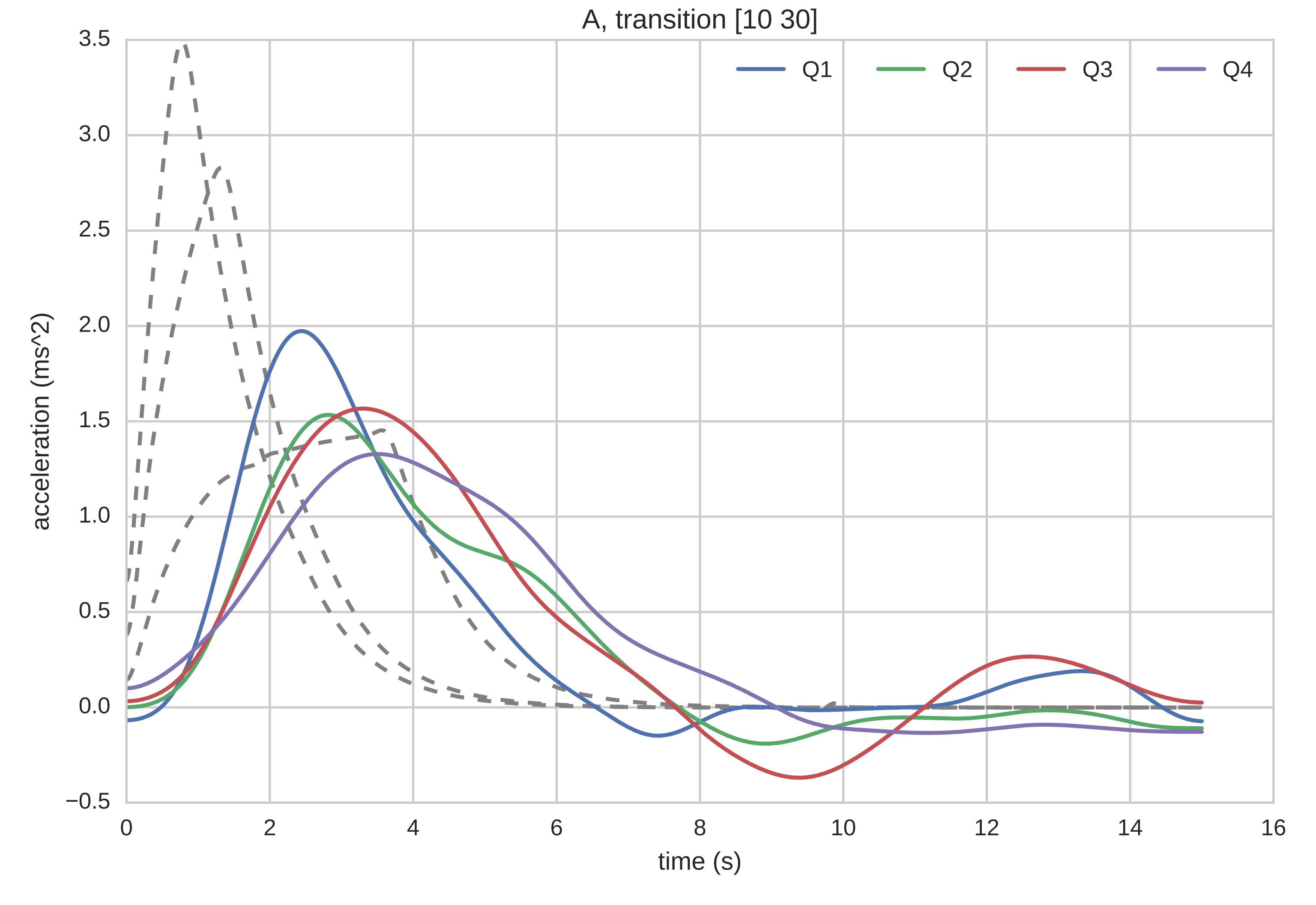
A, transition [10 30]
Q1
Q2
Q3
Q4
acceleration (ms^2)
time (s)
3.5
3.0
2.5
2.0
1.5
1.0
0.5
0.0
−0.5
0
2
4
6
8
10
12
14
16

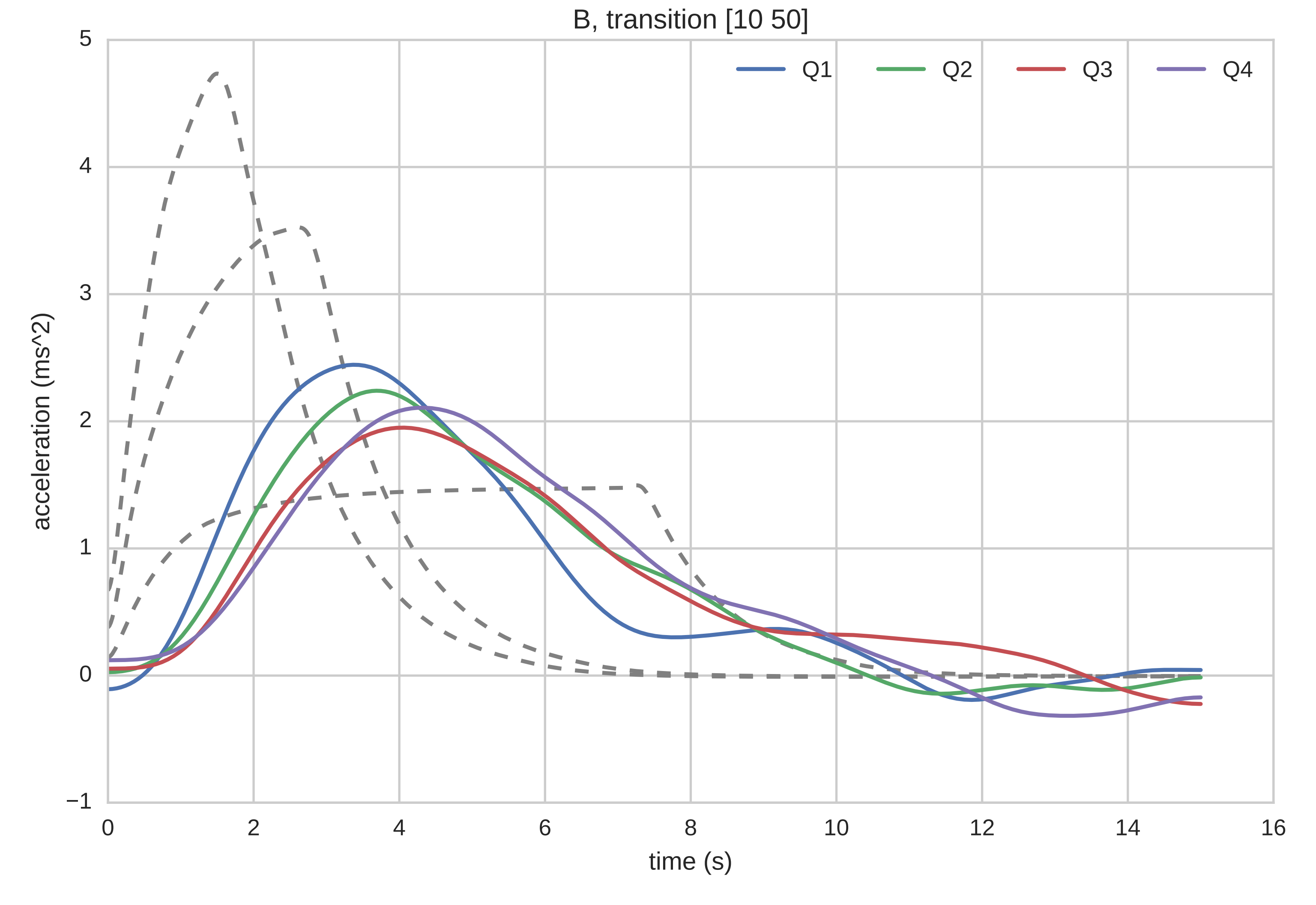
B, transition [10 50]
Q1
Q2
Q3
Q4
acceleration (ms^2)
5
4
3
2
1
0
−1
0
2
4
6
8
10
12
14
16
time (s)

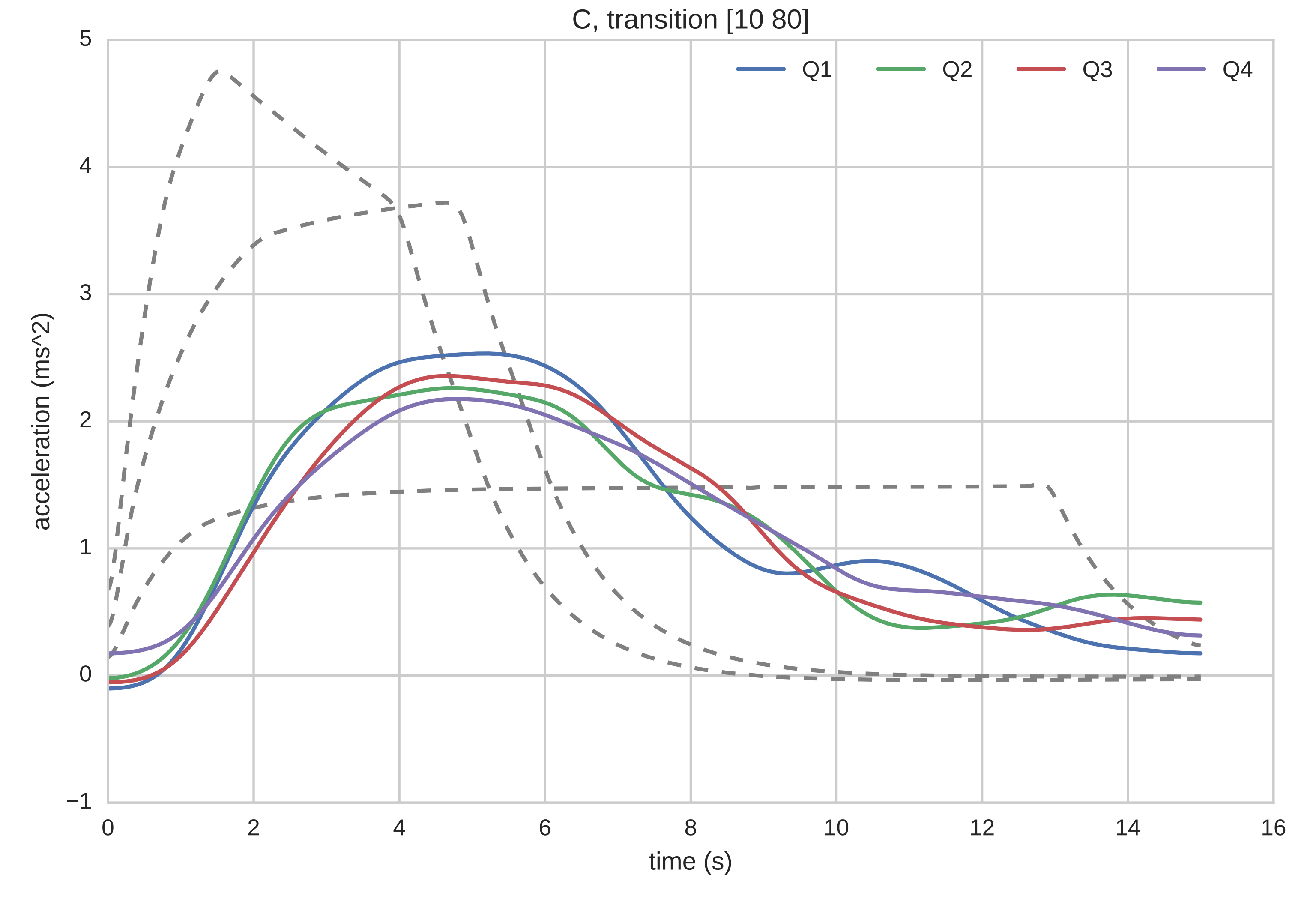

C, transition [10 80]
Q1
Q2
Q3
Q4
acceleration (ms^2)
time (s)
5
4
3
2
1
0
−1
0
2
4
6
8
10
12
14
16

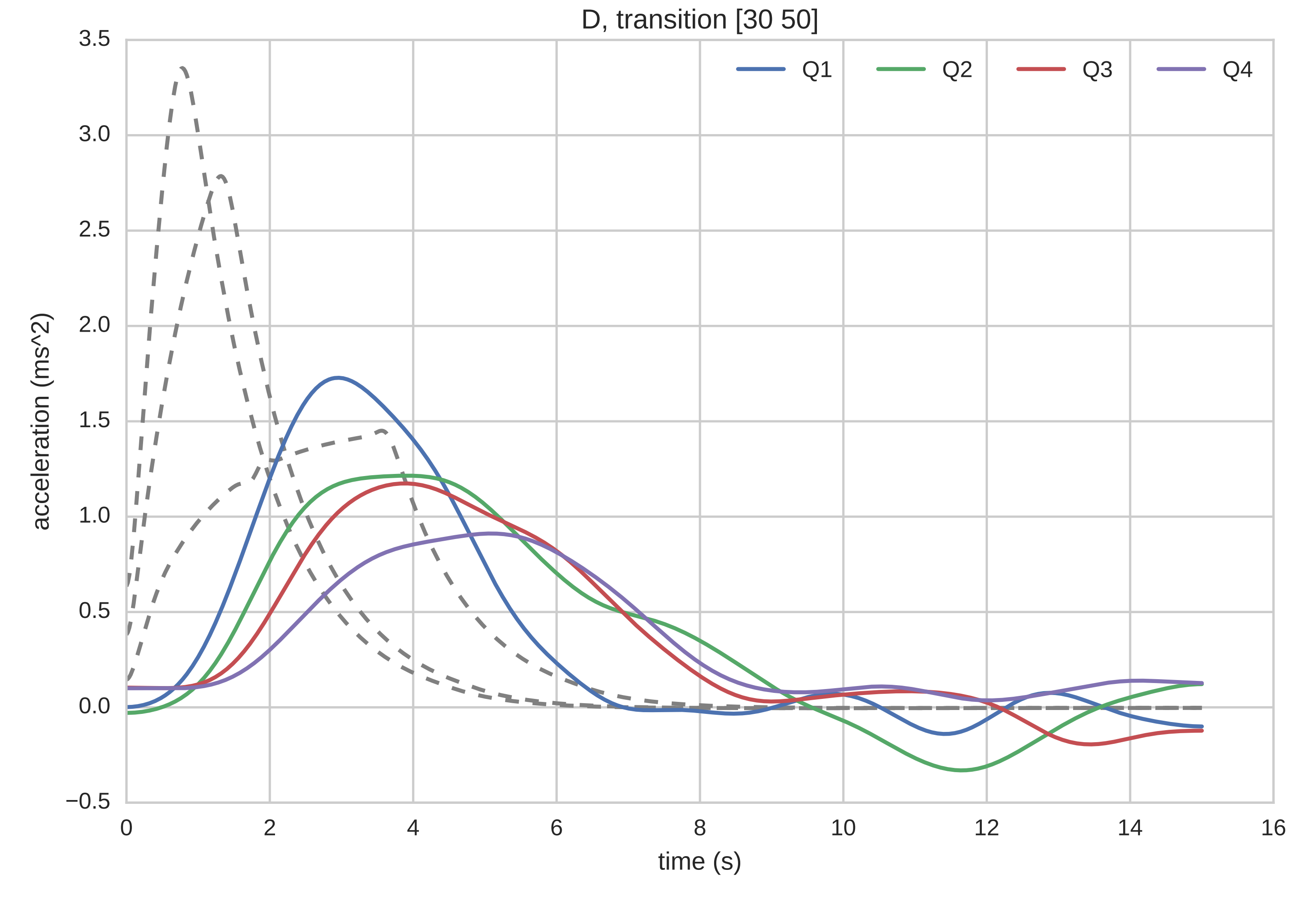
D, transition [30 50]
Q1
Q2
Q3
Q4
3.5
3.0
2.5
2.0
1.5
1.0
0.5
0.0
−0.5
acceleration (ms^2)
0
2
4
6
8
10
12
14
16
time (s)

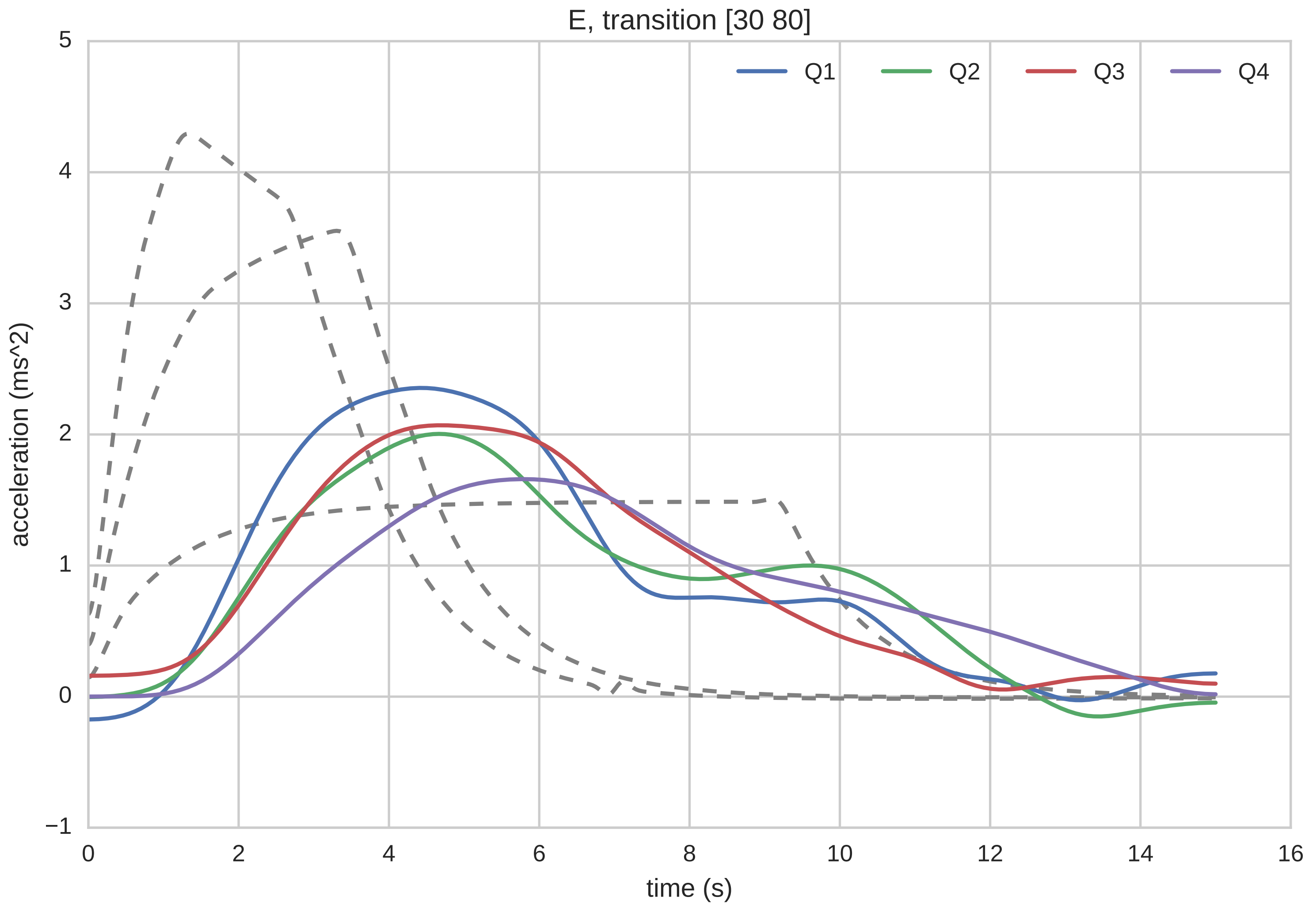
E, transition [30 80]
Q1
Q2
Q3
Q4
acceleration (ms^2)
time (s)
5
4
3
2
1
0
−1
0
2
4
6
8
10
12
14
16

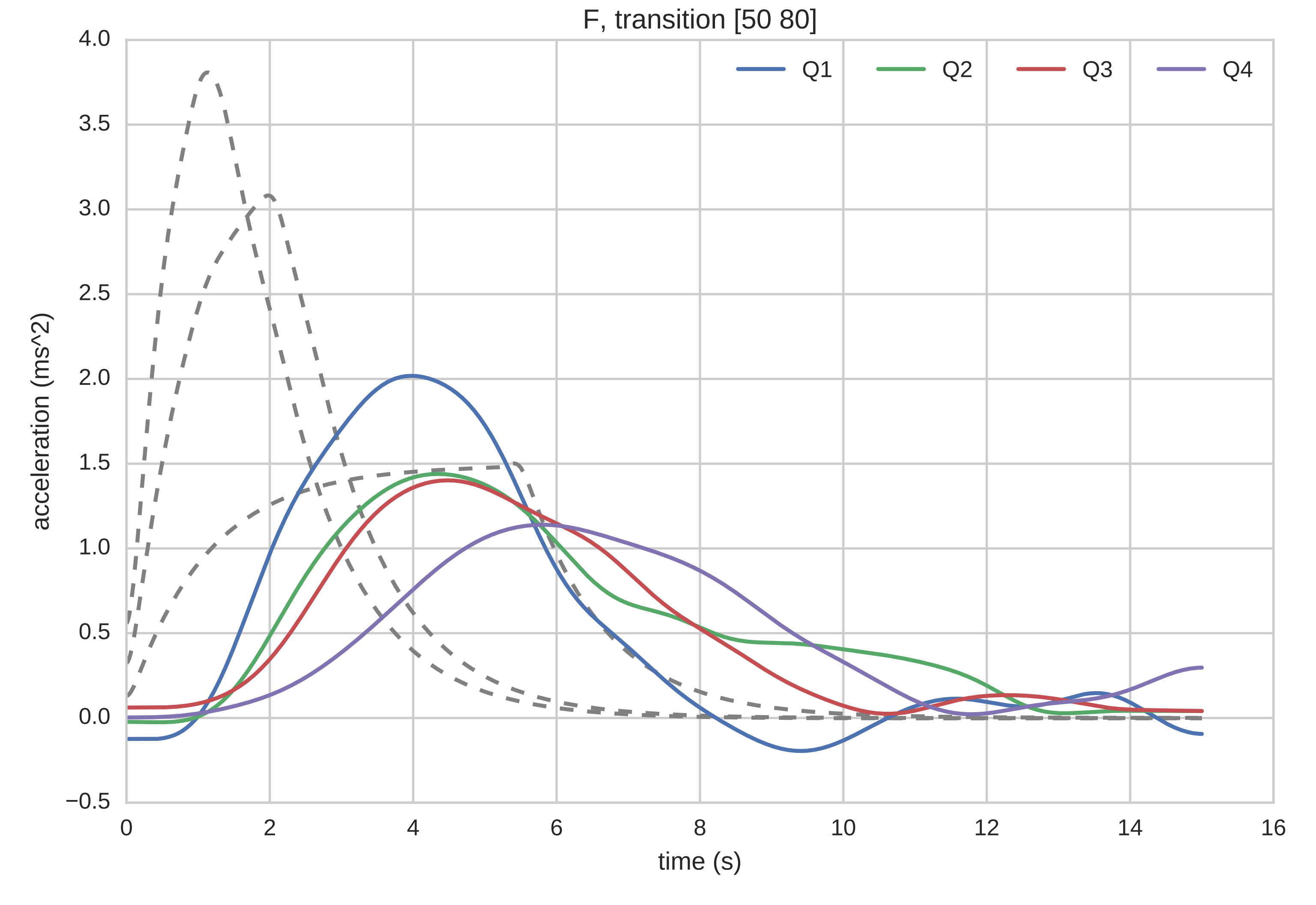

F, transition [50 80]
Q1
Q2
Q3
Q4
acceleration (ms^2)
time (s)
4.0
3.5
3.0
2.5
2.0
1.5
1.0
0.5
0.0
−0.5
0
2
4
6
8
10
12
14
16

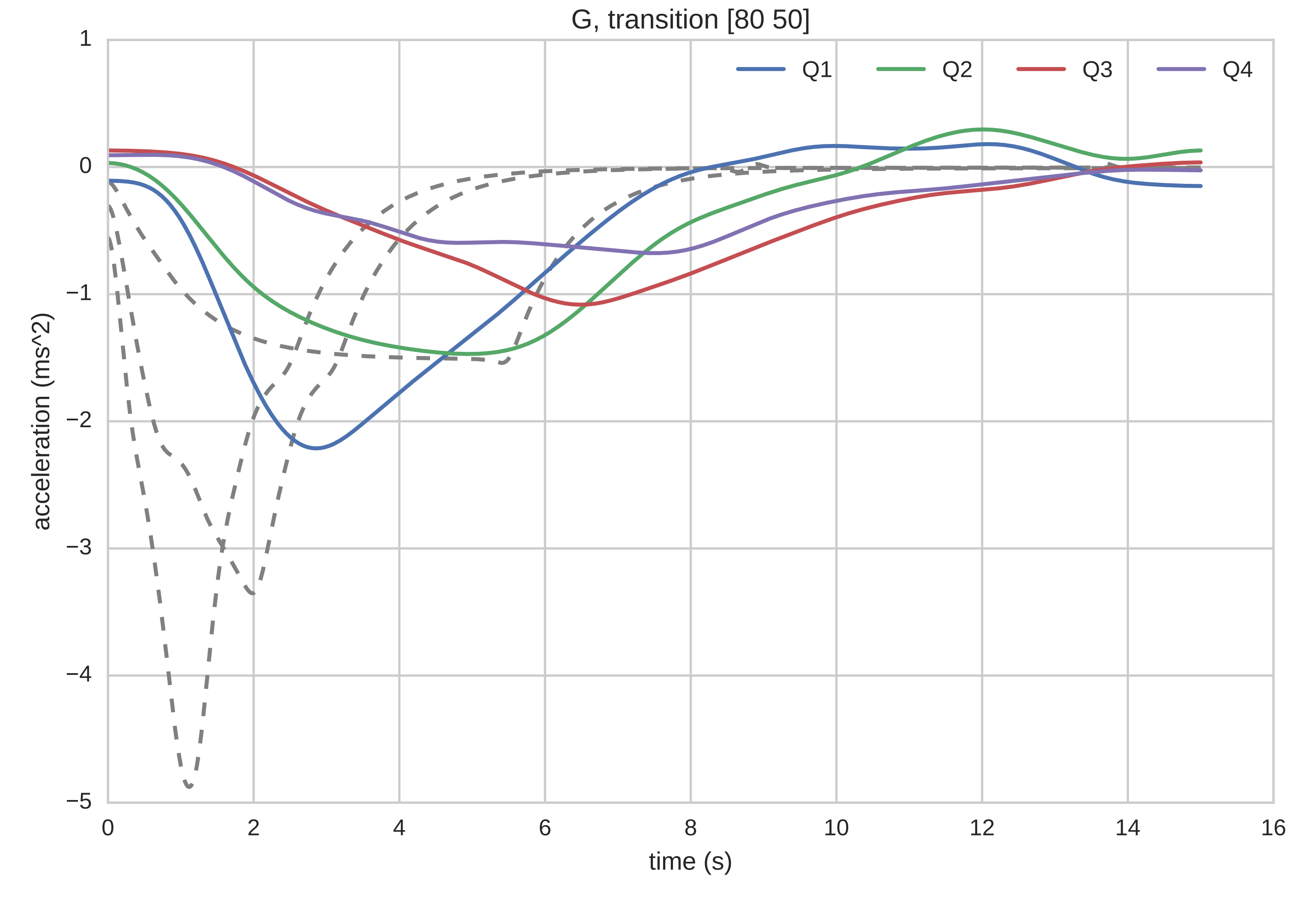

G, transition [80 50]
Q1
Q2
Q3
Q4
acceleration (ms^2)
time (s)
1
0
−1
−2
−3
−4
−5
0
2
4
6
8
10
12
14
16

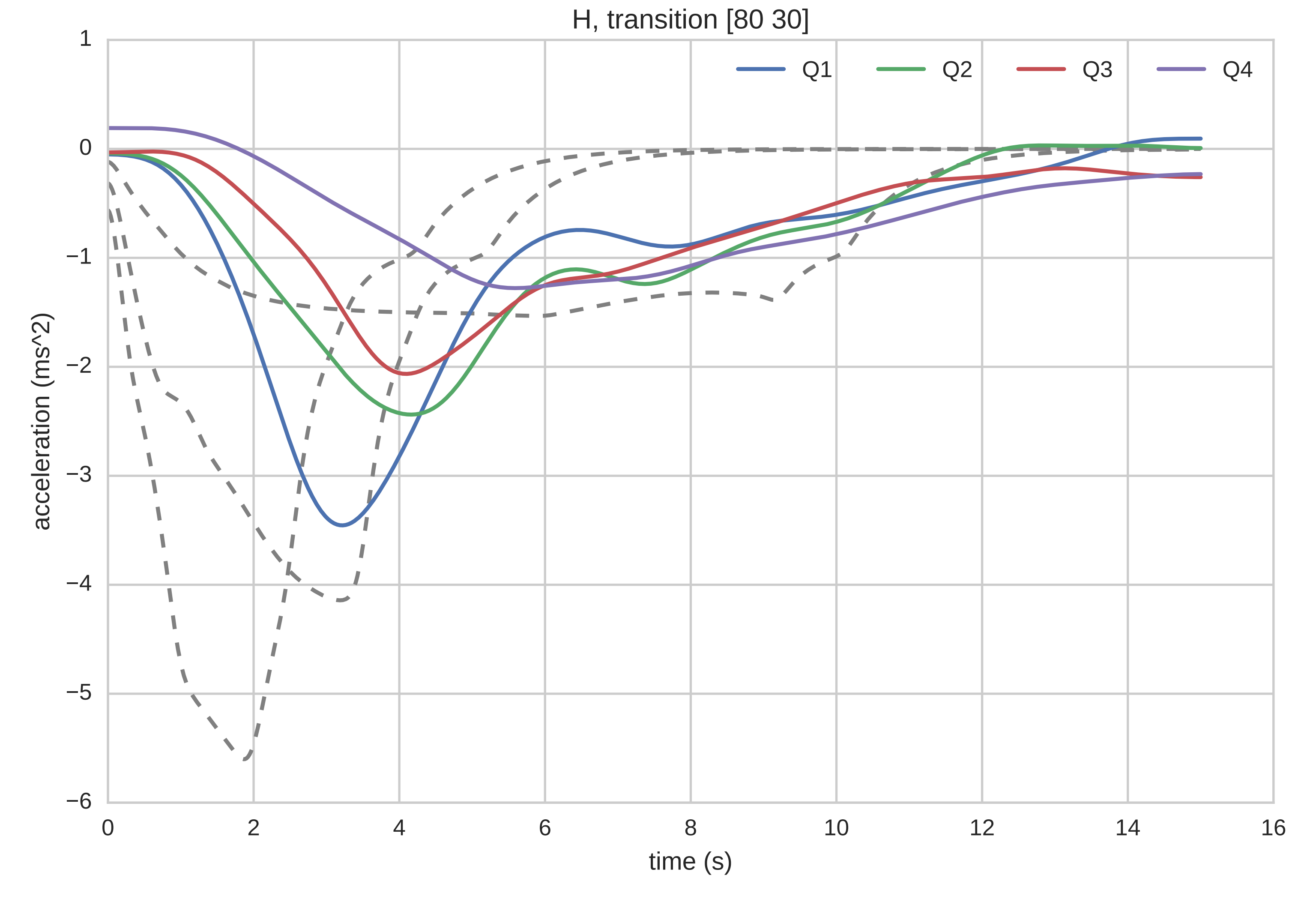
H, transition [80 30]
Q1
Q2
Q3
Q4
acceleration (ms^2)
time (s)
1
0
−1
−2
−3
−4
−5
−6
0
2
4
6
8
10
12
14
16

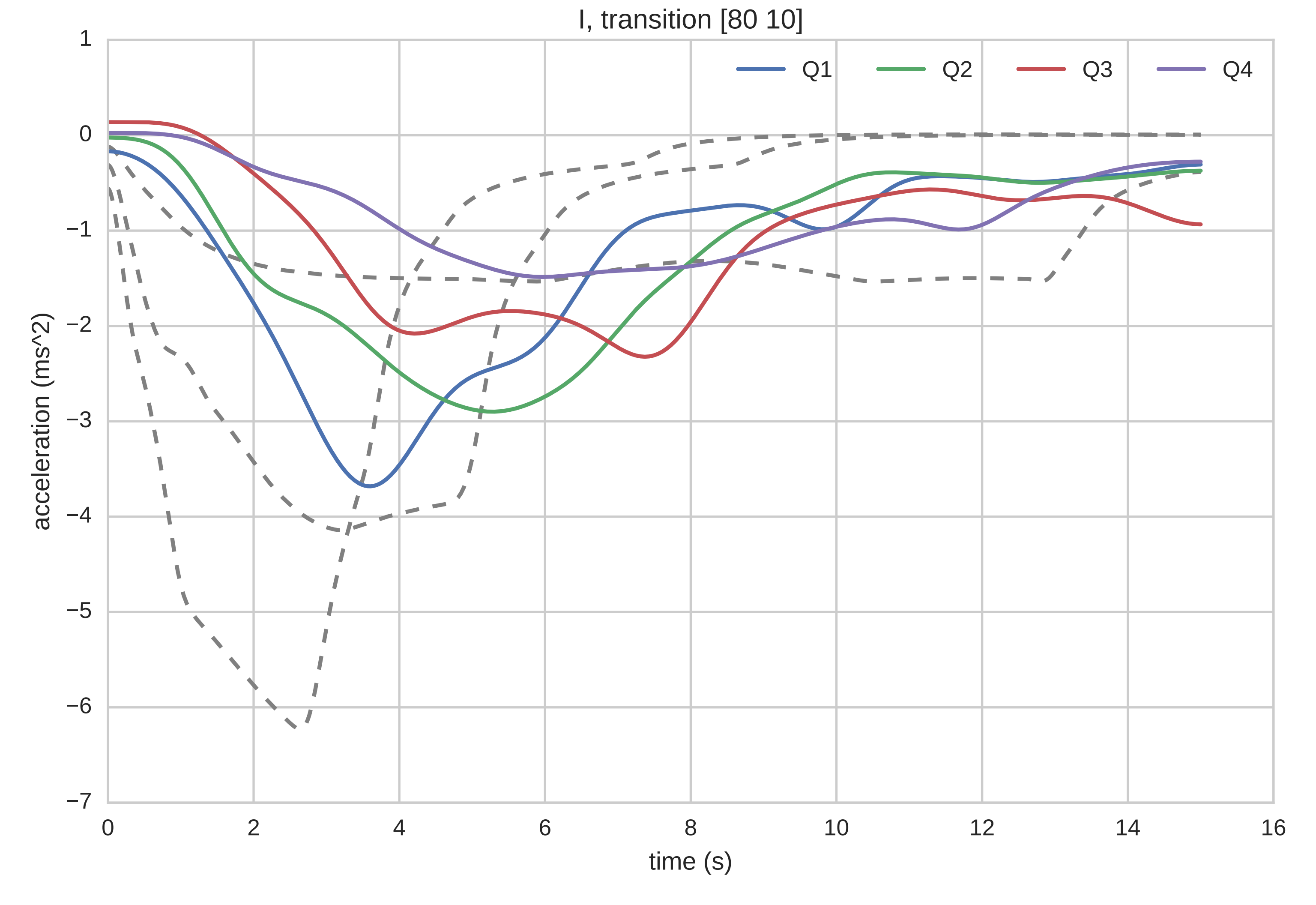

I, transition [80 10]
Q1
Q2
Q3
Q4
acceleration (ms^2)
time (s)
1
0
−1
−2
−3
−4
−5
−6
−7
0
2
4
6
8
10
12
14
16

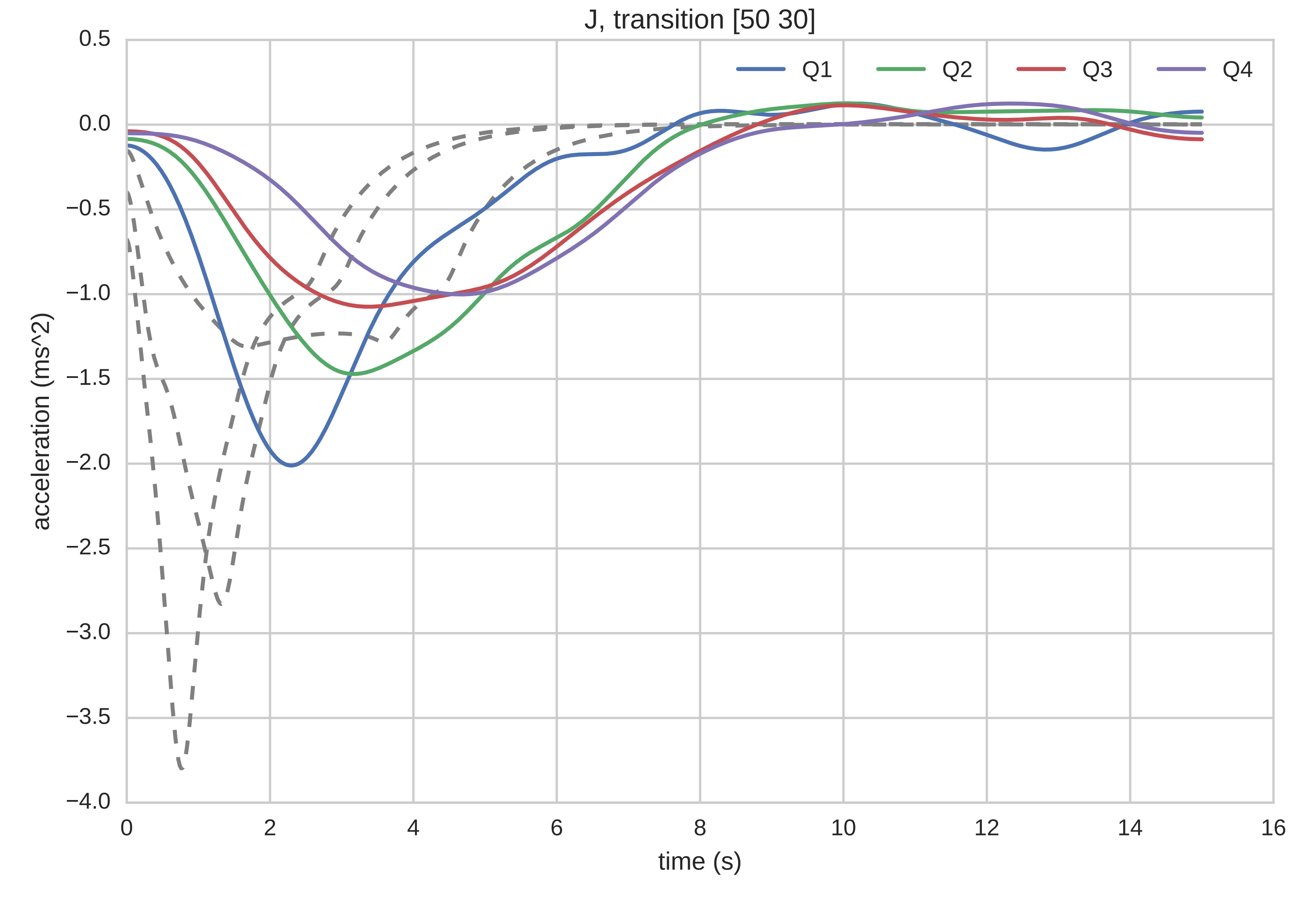
J, transition [50 30]
Q1
Q2
Q3
Q4
acceleration (ms^2)
time (s)
0.5
0.0
−0.5
−1.0
−1.5
−2.0
−2.5
−3.0
−3.5
−4.0
0
2
4
6
8
10
12
14
16

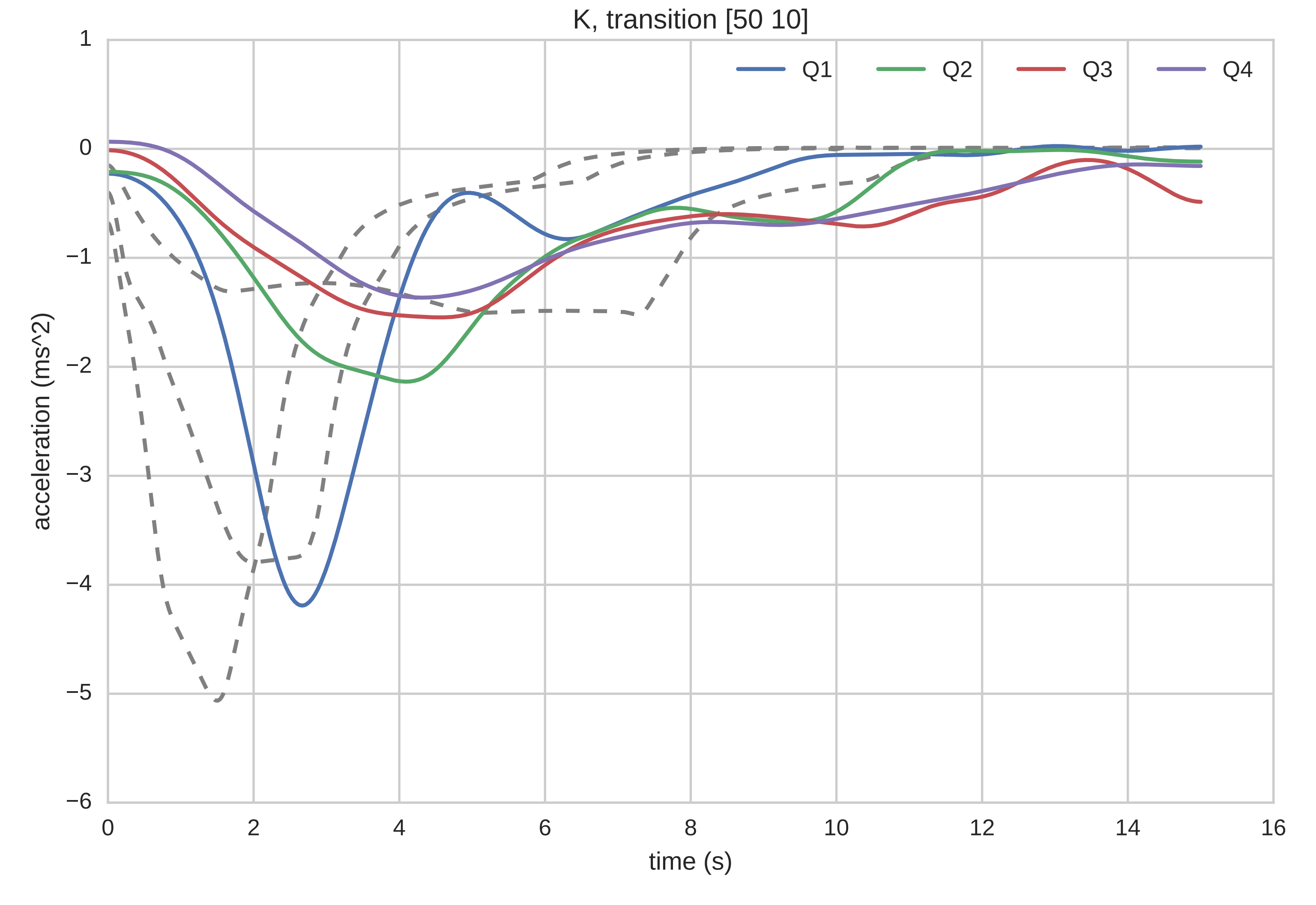

K, transition [50 10]
Q1
Q2
Q3
Q4
acceleration (ms^2)
time (s)
1
0
−1
−2
−3
−4
−5
−6
0
2
4
6
8
10
12
14
16

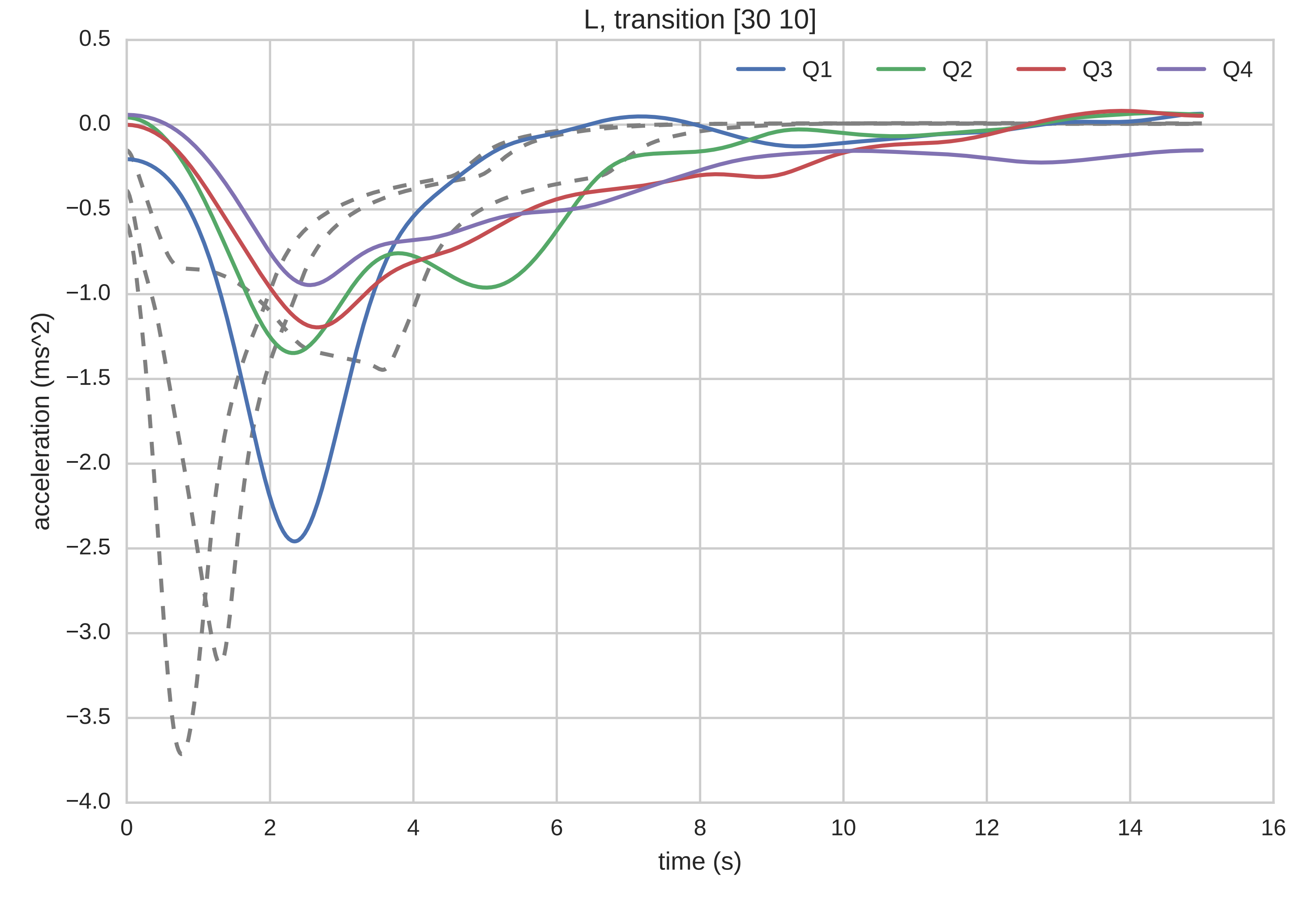
L, transition [30 10]
Q1
Q2
Q3
Q4
acceleration (ms^2)
time (s)
0.5
0.0
−0.5
−1.0
−1.5
−2.0
−2.5
−3.0
−3.5
−4.0
0
2
4
6
8
10
12
14
16
