# Supplementary material for: Trade-off between jerk and time headway as an indicator of driving style
Source: PLoS One. 2017 Oct 17;12(10):e0185856. doi: 10.1371/journal.pone.0185856 (PMC5645088; doi:10.1371/journal.pone.0185856)
Supplement: S2 File — Details of the Principal Component Analysis for variables containing subject averages (N = 15)—mean acceleration, mean jerk and mean time headway. They include eigenvalues, eigenvectors and the loadings for each component. (PDF) [file pone.0185856.s003.pdf]

S2 file. Tables A-C. PCA results.

A. PCA eigenvalues.

	PC 1	PC 2	PC 3
Eigenvalue	2.73	0.22	0.03
Share of variance explained	0.91	0.07	0.01

B. PCA eigenvectors

	PC 1	PC 2	PC 3
Mean THW	-0.56	0.83	0.03
Mean Acceleration	0.59	0.42	-0.69
Mean Jerk	0.59	0.36	0.72

C. PCA loadings (eigenvalues * sqrt(eigenvectors))

	PC 1	PC 2	PC 3
Mean THW	-0.91	0.39	0.006
Mean Acceleration	0.97	0.20	-0.14
Mean Jerk	0.97	0.17	0.14
