# Supplementary figures and images for: Trade-off between jerk and time headway as an indicator of driving style
Source: PLoS One. 2017 Oct 17;12(10):e0185856. doi: 10.1371/journal.pone.0185856 (PMC5645088; doi:10.1371/journal.pone.0185856)

**S1 Fig. Jerk vs. Time Headway – Accelerating and Decelerating case**

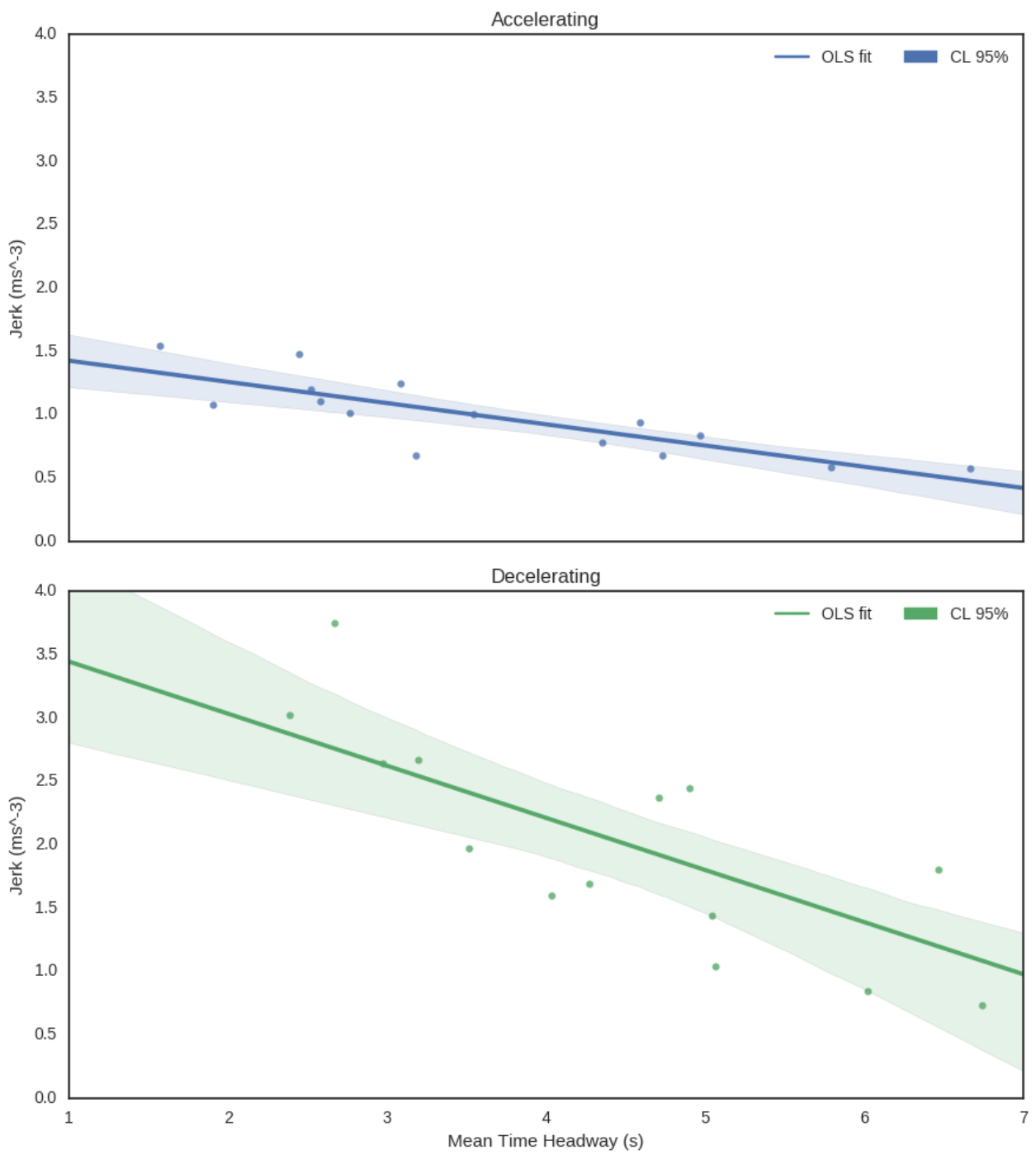

Supplement: S1 Fig — These figures represent the linear fit for the accelerating and decelerating cases for the subject averages of jerk and time headway. The lines are Ordinary Least Squares (OLS) fits along with their confidence intervals at CL 95%. (PDF) [file pone.0185856.s004.pdf]

S2 Fig. Mean jerk and mean time headway with gender

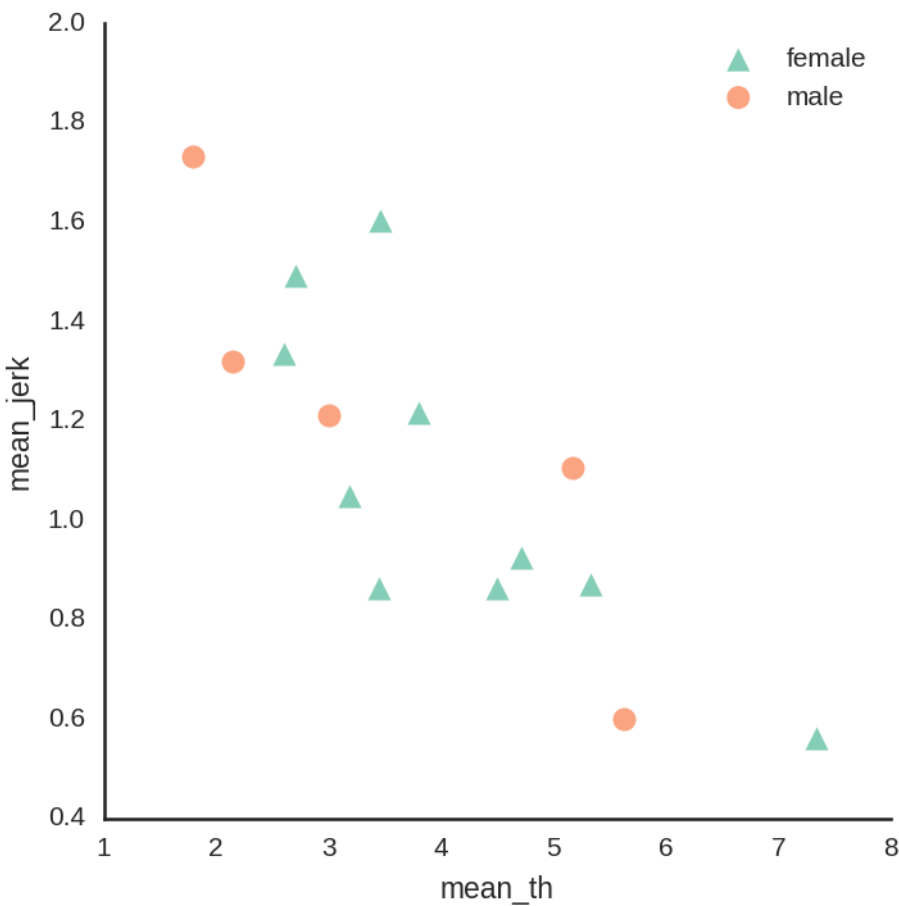

Supplement: S2 Fig — Scatter plot showing mean time headway on the x-axis and mean jerk on the y-axis. Male participants in orange, females in green. (PDF) [file pone.0185856.s005.pdf]

**S3 Fig. First two PCA components with gender**

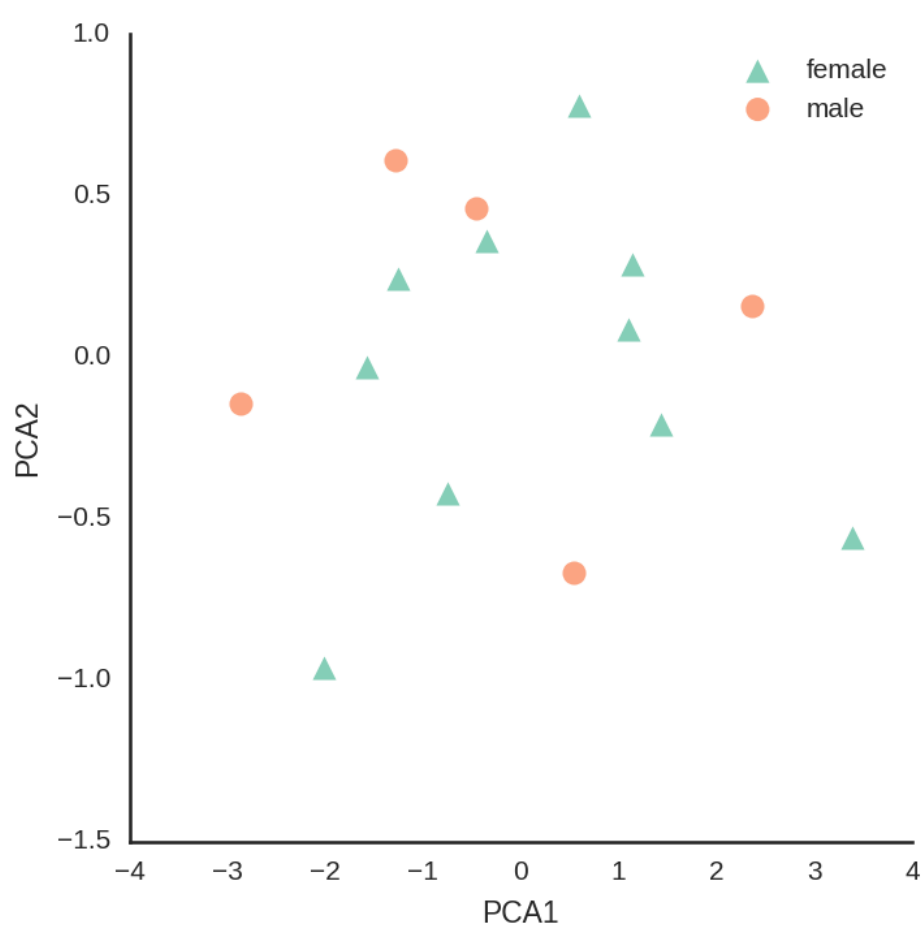

Supplement: S3 Fig — Scatter plot showing the first two PCA components, with gender of the participants differentiated by colour. Should the two groups form distinct clusters, one would be able to see them here. No such pattern arises with this amount of participants. (PDF) [file pone.0185856.s006.pdf]
